# TDAG51 is a crucial regulator of maternal care and depressive-like behavior after parturition

**DOI:** 10.1371/journal.pgen.1008214

**Published:** 2019-06-28

**Authors:** Hyeongseok Yun, Eui-Soon Park, Seunga Choi, Bongjin Shin, Jungeun Yu, Jiyeon Yu, Dulshara Sachini Amarasekara, Sumi Kim, Nari Lee, Jong-Soon Choi, Yongwon Choi, Jaerang Rho

**Affiliations:** 1 Department of Microbiology and Molecular Biology, Chungnam National University, Daejeon, Korea; 2 Division of Life Science, Korea Basic Science Institute, Daejeon, Korea; 3 Department of Pathology and Laboratory Medicine, University of Pennsylvania School of Medicine, Philadelphia, Pennsylvania, United States of America; Cardiff University, UNITED KINGDOM

## Abstract

Postpartum depression is a severe emotional and mental disorder that involves maternal care defects and psychiatric illness. Postpartum depression is closely associated with a combination of physical changes and physiological stress during pregnancy or after parturition in stress-sensitive women. Although postpartum depression is relatively well known to have deleterious effects on the developing fetus, the influence of genetic risk factors on the development of postpartum depression remains unclear. In this study, we discovered a novel function of T cell death-associated gene 51 (TDAG51/PHLDA1) in the regulation of maternal and depressive-like behavior. After parturition, TDAG51-deficient dams showed impaired maternal behavior in pup retrieving, nursing and nest building tests. In contrast to the normal dams, the TDAG51-deficient dams also exhibited more sensitive depressive-like behaviors after parturition. Furthermore, changes in the expression levels of various maternal and depressive-like behavior-associated genes regulating neuroendocrine factor and monoamine neurotransmitter levels were observed in TDAG51-deficient postpartum brain tissues. These findings indicate that TDAG51 plays a protective role against maternal care defects and depressive-like behavior after parturition. Thus, TDAG51 is a maternal care-associated gene that functions as a crucial regulator of maternal and depressive-like behavior after parturition.

## Author summary

Postpartum depression is a severe emotional and mental disease that can affect women typically after parturition. However, the genetic risk factors associated with the development of postpartum depression are still largely unknown. We discovered a novel function of T cell death-associated gene 51 (TDAG51) in the regulation of maternal behavior and postpartum depression. We report that TDAG51 deficiency induces depressive-like and abnormal maternal behavior after parturition. The loss of TDAG51 in postpartum brain tissues induces changes in the expression levels of various maternal and depressive-like behavior-associated genes that regulate the levels of neuroendocrine factors and monoamine neurotransmitters. TDAG51 is a maternal care-associated gene that functions as a crucial regulator of maternal and depressive-like behavior after parturition.

## Introduction

Maternal behavior represents an instinctive pattern of caring for an offspring by a mother [1]. The physical and physiological changes that occur in response to pregnancy and parturition may cause maternal depression, which has deleterious effects on maternal behavior [2]. Postpartum depression is a severe emotional and mental disorder that is becoming a serious social issue; this condition needs adequate attention as it can lead to infant abuse or infanticide caused by deficient or weakened maternal care. It is estimated that approximately 50–80% of women experience a short period of mild depression (baby blues) during a period of pregnancy or after parturition, 10–15% of women have more serious symptoms of postpartum depression, and 0.1–0.2% of women suffer postpartum psychosis [3–7]. The biological risk factors of postpartum depression are not well identified, while many psychosocial factors, such as a personal history of psychiatric illness, poor partner/social/financial support and alcohol/drug abuse, and many obstetric factors, such as unplanned pregnancy, pregnancy complications and delivery modes, have been historically well studied [5, 8–11]. In particular, the genetic risk factors underlying postpartum depression remain largely unknown, although a few gene functions or polymorphisms theoretically linked to an increased risk of postpartum depression have been reported [4, 12–16]. Thus, to better understand the underlying causes of postpartum depression based on genetic risk factors, identifying genes or gene functions possibly associated with postpartum depression is necessary.

T cell death-associated gene 51 (TDAG51), which is also known as pleckstrin homology-like domain (PHL) family A member 1 (PHLDA1), contains an N-terminal PHL domain, a C-terminal proline-glutamine (PQ)-repeat domain and a proline-histidine (PH)-repeat domain and functions as a transcription factor [17]. We have previously shown that TDAG51-deficient (TDAG51^-/-^) mice have no developmental abnormalities and do not exhibit a failure of secondary lymphoid organs and alterations in T cell apoptosis [18]. Interestingly, previous studies conducted by our group and other researchers have shown that TDAG51 is highly inducible in response to diverse cellular stresses, including endoplasmic reticulum stress, heat shock and oxidative stress [19–21]. TDAG51 is ubiquitously expressed in most organs and is present at high levels in the brain, thymus, lung and liver relative to other tissues [17]. Interestingly, previous studies have reported that TDAG51 expression is significantly altered in the anterior temporal neocortex in patients with intractable epilepsy, immature rat brain tissues following lipopolysaccharide exposure and the spinal cord in a mouse model of amyotrophic lateral sclerosis [22–24]. Furthermore, TDAG51 is strongly upregulated in the hippocampus and cerebral cortex in response to chronic mild stress exposure and the hippocampus after transient forebrain ischemia [25, 26]. Because TDAG51 is expressed in brain tissues at relatively high levels and is transiently regulated by intrinsic/extrinsic stimuli, such as injury, infection and stress, TDAG51 may play an important role in the brain. In our current study, an unacceptably high level of pup mortality and a deficit in maternal care during the early postnatal period were observed in the breeding cages of the TDAG51^-/-^ mice. Thus, based on our observations and previous studies, we hypothesize that TDAG51 deficiency may elicit depressive-like behavior and maternal care defects after parturition.

Previous studies have revealed that physiological changes occur in the levels of several neuroendocrine factors, including polypeptide hormones, gonadal steroids and hypothalamic-pituitary-adrenal (HPA) axis hormones, as a result of genetic alterations or stress responses and that these changes are closely linked to postpartum depression and maternal behavior [1, 27–31]. In such studies, the functions and regulatory roles of neuroendocrine systems, such as the oxytocin (OXT)/OXT receptor (OXTR), estrogen/estrogen receptor 1 (ESR1), arginine vasopressin (AVP)/AVP receptor 1a (AVPR1A), corticotropin-releasing hormone (CRH)/CRH receptor 1 (CRHR1) and the prolactin (PRL)/PRL receptor systems, in maternal behavior and depression have been elucidated [1, 32–36]. Changes in the levels of neurotransmitters or neurochemicals, including serotonin, epinephrine, norepinephrine, GABA and nitrous oxide, have also been implicated in depression and maternal behavior [1, 29]. Moreover, alterations in the levels of monoaminergic neurotransmitters, such as serotonin, dopamine and norepinephrine, as a result of single-nucleotide polymorphisms in the serotonin transporter, monoamine oxidase A, tryptophan hydroxylase (TPH) and catechol-O-methyltransferase have been evaluated to elucidate their contributions to the development of postpartum depression and anxiety [4, 12–15]. Thus, given the pivotal importance of neuroendocrine systems and neurotransmitters in postpartum depression and maternal behavior, studying the possible links between postpartum depression and maternal behavior at the genetic level in response to alterations in the levels of single genes affecting the regulation of neuroendocrine systems or neurotransmitter levels is important [4, 37]. However, thus far, few studies have addressed this topic [1, 2, 4], and the genetic factors contributing to postpartum depression and maternal behavior remain poorly understood.

In the current study, we examined whether TDAG51 deficiency is linked to the development of abnormal maternal and depressive-like behavior after parturition. We examined the survival rates of offspring in the breeding cages of TDAG51^-/-^ mice and investigated maternal, anxiety-like and depressive-like behaviors in TDAG51^-/-^ dams. Finally, we analyzed the transcriptional alterations in the regulators of neuroendocrine systems or neurotransmitter levels in the brain tissue of TDAG51^-/-^ dams. In this study, we demonstrated that TDAG51^-/-^ dams exhibited maternal care defects and enhanced susceptibility to depressive-like behavior after parturition.

## Results

### Survival of pups born to TDAG51^-/-^ dams is significantly reduced after parturition

**Fig 1:**
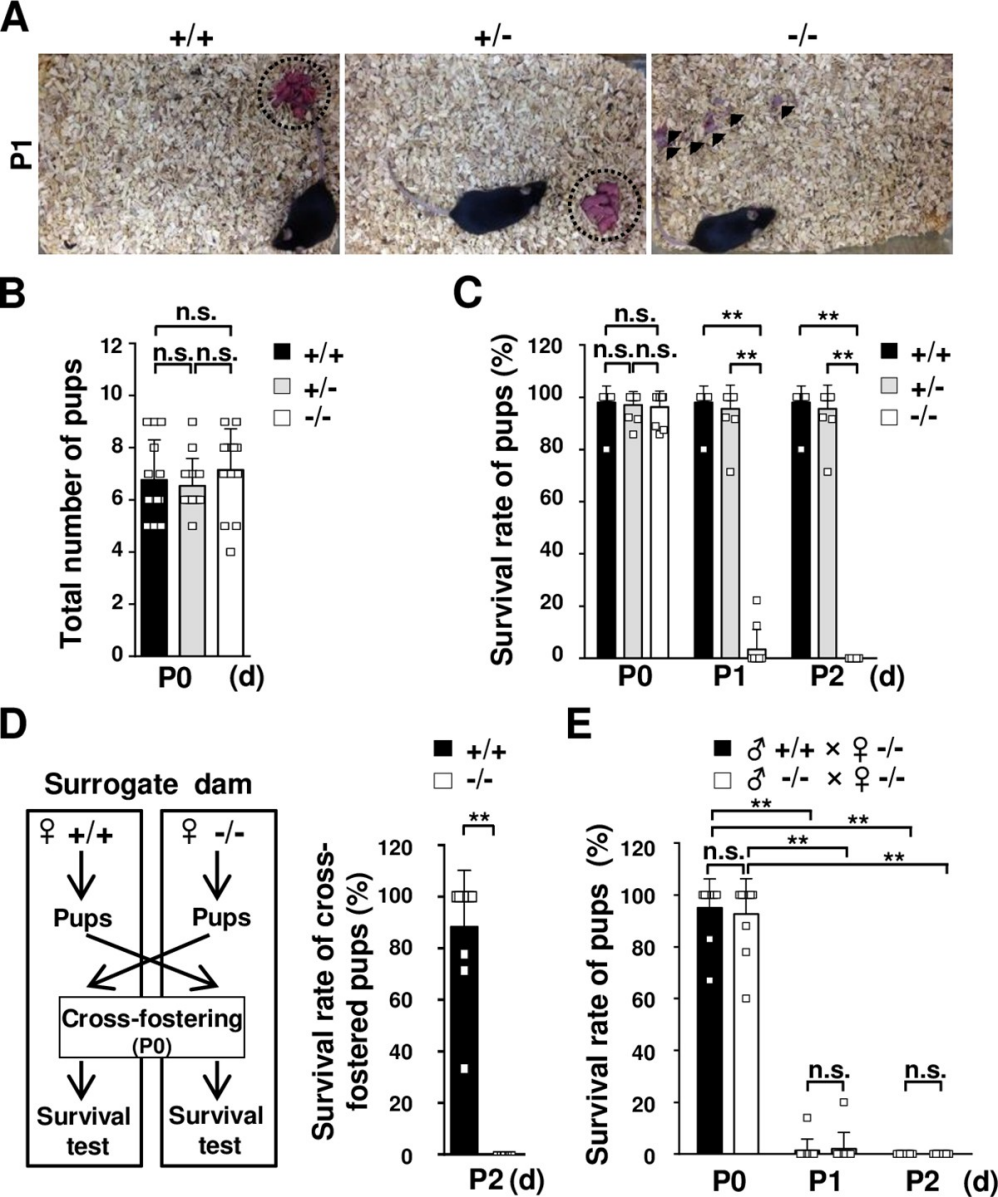
The survival rate of pups born to TDAG51^-/-^ dams is reduced during the early postpartum period. (A) Survival of pups born to TDAG51^-/-^ dams. The photographs were obtained on postnatal day 1 (P1). Arrowheads indicate dead pups. Dotted circles indicate pups gathered in a nest. +/+, TDAG51^+/+^ dams. +/-, TDAG51^+/-^ dams. -/-, TDAG51^-/-^ dams. (B) The average number of pups born to TDAG51^-/-^ dams. The number of pups per dam was analyzed on P0. Black bar (+/+), TDAG51^+/+^ dams. Gray bar (+/-), TDAG51^+/-^ dams. White bar (-/-), TDAG51^-/-^ dams. (C) The survival rate of pups born to TDAG51^-/-^ dams. Pup survival was measured from P0 to P2 in the absence of male mating partners. (D) The survival rate of pups with surrogate TDAG51^-/-^ dams. A schematic diagram of the cross-fostering experiment between TDAG51^+/+^ and TDAG51^-/-^ dams is shown in the left panel. Pups born to TDAG51^+/+^ and TDAG51^-/-^ dams were interchanged on P0, and the survival of the cross-fostered pups with their surrogate dams was analyzed on P2. Black bar (+/+), TDAG51^+/+^ surrogate dams. White bar (-/-), TDAG51^-/-^ surrogate dams. (E) The effect of male mating partners on the survival of pups born to TDAG51^-/-^ dams. Pup survival was analyzed from P0 to P2 in the presence of the male mating partner. Black bar, TDAG51^-/-^ dams with TDAG51^+/+^ male mice. White bar, TDAG51^-/-^ dams with TDAG51^-/-^ male mice. **p<0.01. n.s., not significant.

TDAG51^-/-^ mice are healthy and have no gross developmental abnormalities [18]. However, we observed that the number of surviving pups in the home cages of the TDAG51^-/-^ dams was reduced on postnatal day 1 (P1) (Fig 1A). The dead bodies of the newborn pups or their body parts resulting from the cannibalism of the dead pups by the TDAG51^-/-^ dams were scattered around the home cages, while the pups in the TDAG51^+/+^ or TDAG51^+/-^ dam cages were well fostered (Fig 1A). The TDAG51^-/-^ dams did not exhibit parturition problems (*F*_2, 36_ = 0.64, *p* = 0.54) (Fig 1B) and showed normal development in terms of the mammary glands and milk production (S1A Fig). In addition, the pups born to the TDAG51^-/-^ dams did not have postpartum suckling problems (S1B Fig). These results indicate that TDAG51^-/-^ dams may have a severe maternal care defect toward their pups after parturition.

To investigate this possibility, we analyzed the survival rate of the pups born to the TDAG51^-/-^ dams during the early postpartum period by a two-way analysis of variance (ANOVA) with repeated measures, followed by a *post hoc* analysis. On P0, the pup survival in the TDAG51^-/-^ dam cages did not differ from that in the TDAG51^+/+^ (*F*_2, 27_ = 378, *p* = 0.82) and TDAG51^+/-^ dam cages (*F*_2, 27_ = 378, *p* = 0.97) (Fig 1C). However, the survival rate of the pups in the TDAG51^-/-^ dam cages was decreased to 3.5±2.4% (*F*_2, 27_ = 378, *p*<0.01) compared to that in the TDAG51^+/+^ and TDAG51^+/-^ dam cages (98.0±2.0% and 95.5±2.9%, respectively) on P1, and no surviving pups were observed in the TDAG51^-/-^ dam cages on P2 (Fig 1C). To further test the effect of the TDAG51^-/-^ dams on pup survival, we conducted a pup cross-fostering experiment between the home cages of the TDAG51^+/+^ and TDAG51^-/-^ dams. To test the survival rate of the cross-fostered pups, the pups born to the TDAG51^-/-^ dams were immediately transferred to the nests of TDAG51^+/+^ surrogate dams on P0, while the pups born to the TDAG51^+/+^ dams were transferred to the nests of TDAG51^-/-^ surrogate dams as illustrated in Fig 1D (left). After 2 days (P2), approximately 88.3±7.0% of the transferred TDAG51^-/-^ pups had survived in the TDAG51^+/+^ surrogate dam cages, but all transferred TDAG51^+/+^ pups died in the TDAG51^-/-^ surrogate dam cages (*t*_18_ = 12.7, *p*<0.001) (Fig 1D (right)). To exclude the effect of male mating partners on pup survival, we examined the survival rate of the pups in the TDAG51^-/-^ dam cages in the presence of TDAG51^-/-^ or TDAG51^+/+^ male mating partners. Similar to the results shown in Fig 1C, we observed very low survival rates (1.4±1.4% or 2.0±2.0%) on P1 or no surviving pups on P2 in the TDAG51^-/-^ dam cages regardless of the genotype of the male mating partner in the TDAG51^-/-^ dam cages as analyzed by a two-way ANOVA with repeated measures (*F*_1, 18_ = 0.13, *p* = 0.72) (Fig 1E). Taken together, these results suggest that TDAG51 deficiency elicits abnormal maternal care toward pups during the early postpartum period.

### TDAG51 deficiency elicits abnormal maternal behavior after parturition

**Fig 2:**
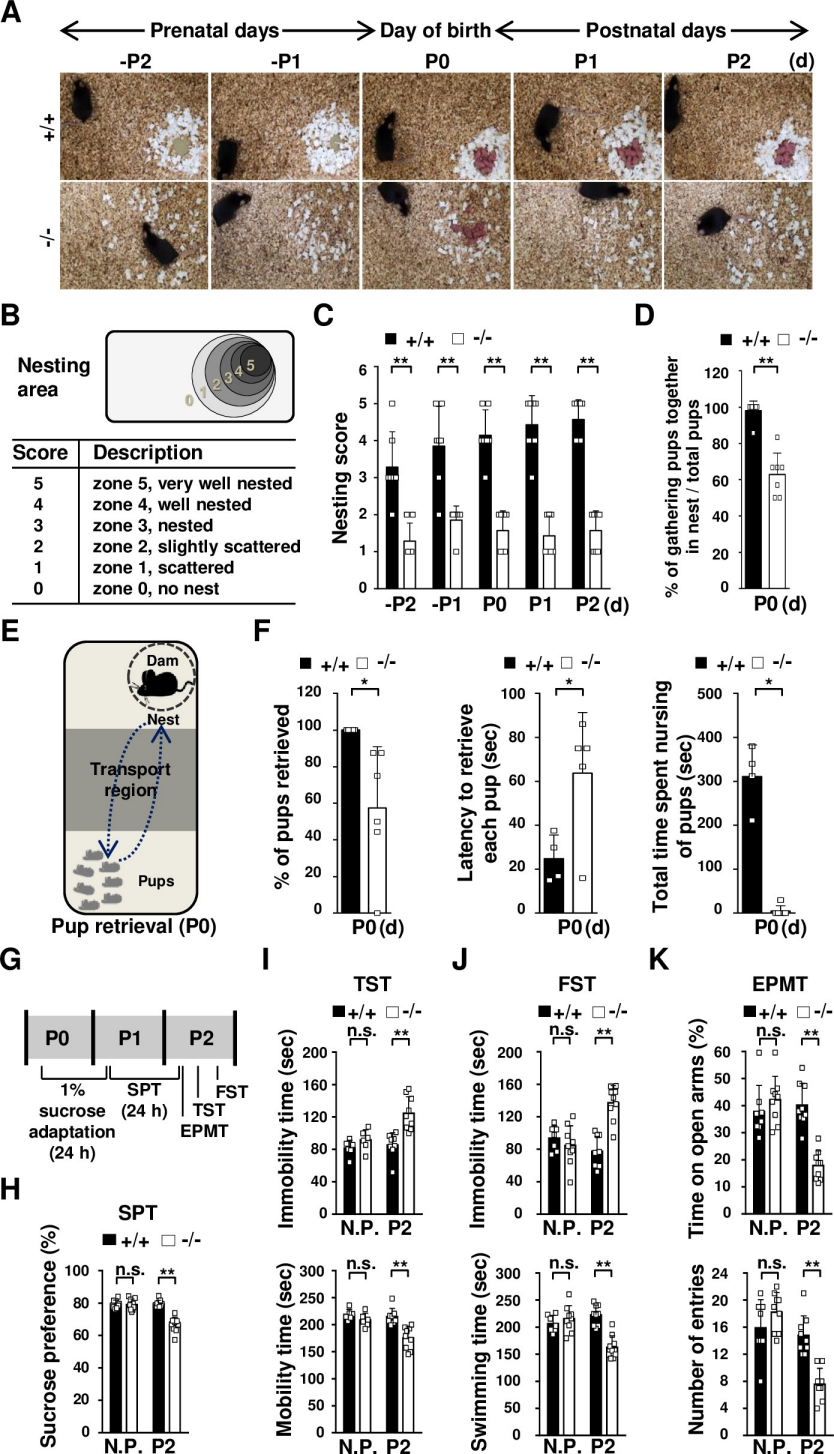
TDAG51 deficiency elicits abnormal maternal behavior and depressive-like behavior after parturition. (A) Reduced nest building ability in TDAG51^-/-^ female mice. TDAG51^+/+^ or TDAG51^-/-^ female mice with vaginal plugs were separated from their male mating partners and given nesting material on prenatal day 3 (-P3). Photographs of nest building were obtained from -P2 to P2. +/+, TDAG51^+/+^ dams. -/-, TDAG51^-/-^ dams. (B) Diagram of the measurement of the nest building score. Nesting scores (0–5) indicate the extent of nest building. (C) TDAG51^-/-^ female mice have impaired nest building abilities. Black bar (+/+), TDAG51^+/+^ dams. White bar (-/-), TDAG51^-/-^ dams. (D) Percentage of pups gathered in a nest. The number of pups gathered in a nest expressed as a percentage of the total number of neonatal pups measured on P0. (E) Diagram of pup retrieval analysis. (F) Lower pup retrieval by TDAG51^-/-^ dams. Each dam was separated from their pups in a home cage for 5 min. The number of retrieved pups was determined by analyzing a 10-minute video-recording. Left panel, the percentage of retrieved pups per dam. Middle panel, latency to retrieve each pup by the TDAG51^-/-^ dams. Right panel, impaired nursing of the retrieved pups by the TDAG51^-/-^ dams. (G) Diagram of the depressive-like and anxiety-like behavior tests. Nonpregnant (N.P.) female mice or postpartum dams were subjected to 1% sucrose adaptation on P0 for 24 h. Then, 1% sucrose and water were supplied to the mice in their home cages on P1. An SPT was performed on P2; then, the mice were subjected to an EPMT, a TST and an FST in an orderly manner on P2. (H) SPT. Sucrose preference was measured for 24 h. (I) In the TST, the mobility time and immobility time over a 5-min period were analyzed. (J) In the FST, the swimming time and immobility time over a 5-min period were analyzed. (K) In the EPMT, the female mice were exposed to an elevated plus-maze, and the time spent in the open arms and the number of entries were determined over the 10-min test period. *p<0.05. **p<0.01. n.s., not significant.

Subsequently, we investigated whether TDAG51 deficiency is associated with maternal behavior during the early postpartum period. In the nest building test, pregnant mice were given cotton nesting material on prenatal day 3 (-P3), and their nest building abilities during the prenatal, parturition and postnatal periods from -P2 to P2 were evaluated. We observed that the TDAG51^-/-^ mice had poorly organized nests, while the TDAG51^+/+^ mice built nearly perfect nests (Fig 2A). Then, we qualitatively ranked the nest building ability as nesting scores on a scale from 0–5 (5 being the best) as illustrated in Fig 2B. Based on the nesting scores, the two-way ANOVA with repeated measures revealed that the nest building ability of the TDAG51^-/-^ mice was significantly worse (*F*_1, 12_ = 121, *p*<0.01) than that of the TDAG51^+/+^ mice (Fig 2C). Moreover, the pups born to the TDAG51^-/-^ dams were scattered around the home cages on P0, resulting in a reduction in the percentage of gathered pups (~35.1% reduction), while the pups born to the TDAG51^+/+^ dams were well gathered in the nest (*t*_12_ = 7.2, *p*<0.01) (Fig 2D). The results shown in Fig 2A–2D indicate that the TDAG51^-/-^ dams may have impaired maternal care. To address this possibility, we performed a pup retrieval test on P0 as illustrated in Fig 2E. The percentage of retrieved pups in the cages with the TDAG51^-/-^ dams was significantly lower (~42.6% reduction (*t*_11_ = 2.46, *p*<0.05)) than that in the cages with the TDAG51^+/+^ dams (Fig 2F (left) and S1 Table). In addition, the TDAG51^-/-^ dams displayed a longer retrieving latency (~2.3-fold (*t*_11_ = 2.64, *p*<0.05)) and spent significantly less time nursing the pups (*t*_11_ = 10.5, *p*<0.01) than the TDAG51^+/+^ dams (Fig 2F (middle and right) and S1 Table). Taken together, these results indicate that TDAG51 deficiency results in abnormal maternal behavior.

### TDAG51^-/-^ dams have enhanced susceptibility to depressive-like behavior after parturition

To investigate whether TDAG51 deficiency affects the occurrence of depressive-like behavior after parturition, we performed depressive-like behavior tests, including the sucrose preference test (SPT), tail suspension test (TST) and forced swim test (FST), and an elevated plus-maze test (EPMT) was used to measure anxiety-like behavior. As illustrated in Fig 2G, in the SPT, the TDAG51^+/+^ and TDAG51^-/-^ dams were adapted to 1% sucrose water for 24 h after parturition (P0) and then given 24-h access to a 1% sucrose solution and tap water on P1. The TDAG51^-/-^ dams exhibited a significantly lower (~13.0% reduction (*F*_3, 32_ = 33.0, *p*<0.01)) sucrose preference than the TDAG51^+/+^ dams on P2, while no difference was observed between the TDAG51^+/+^ and TDAG51^-/-^ nonpregnant female mice (Fig 2H). As illustrated in Fig 2G, the following behavioral tests were conducted on P2 after the SPT in sequential order with a time interval of 4 h or 6 h for each behavioral test to minimize stress induced by the previous behavioral test as previously described [38, 39]: EPMT, TST and FST. During the TST, in the TDAG51^-/-^ dams, the immobility time was longer and the mobility time was shorter than those in the TDAG51^+/+^ dams (*F*_3, 32_ = 12.5, *p*<0.01); in contrast, the nonpregnant TDAG51^+/+^ and TDAG51^-/-^ female mice did not show any significant differences in the TST (Fig 2I). Consistent with these results, the TDAG51^-/-^ dams showed a longer immobility time and a shorter swimming time in the FST (*F*_3, 32_ = 15.6, *p*<0.01) (Fig 2J). Subsequently, we performed an EPMT to measure anxiety-like behavior because postpartum depression is often accompanied by anxiety-like phenotypes [40]. The TDAG51^-/-^ dams spent significantly less time in the open arms (*F*_3, 32_ = 17.2, *p*<0.01) and showed significantly fewer entries (*F*_3, 32_ = 19.4, *p*<0.01) into the open arms than the TDAG51^+/+^ dams (Fig 2K), suggesting that TDAG51^-/-^ dams exhibit increased anxiety-like phenotype compared to TDAG51^+/+^ dams. Taken together, these results indicate that TDAG51^-/-^ dams are more susceptible to depressive-like and anxiety-like behavior after parturition.

**Fig 3:**
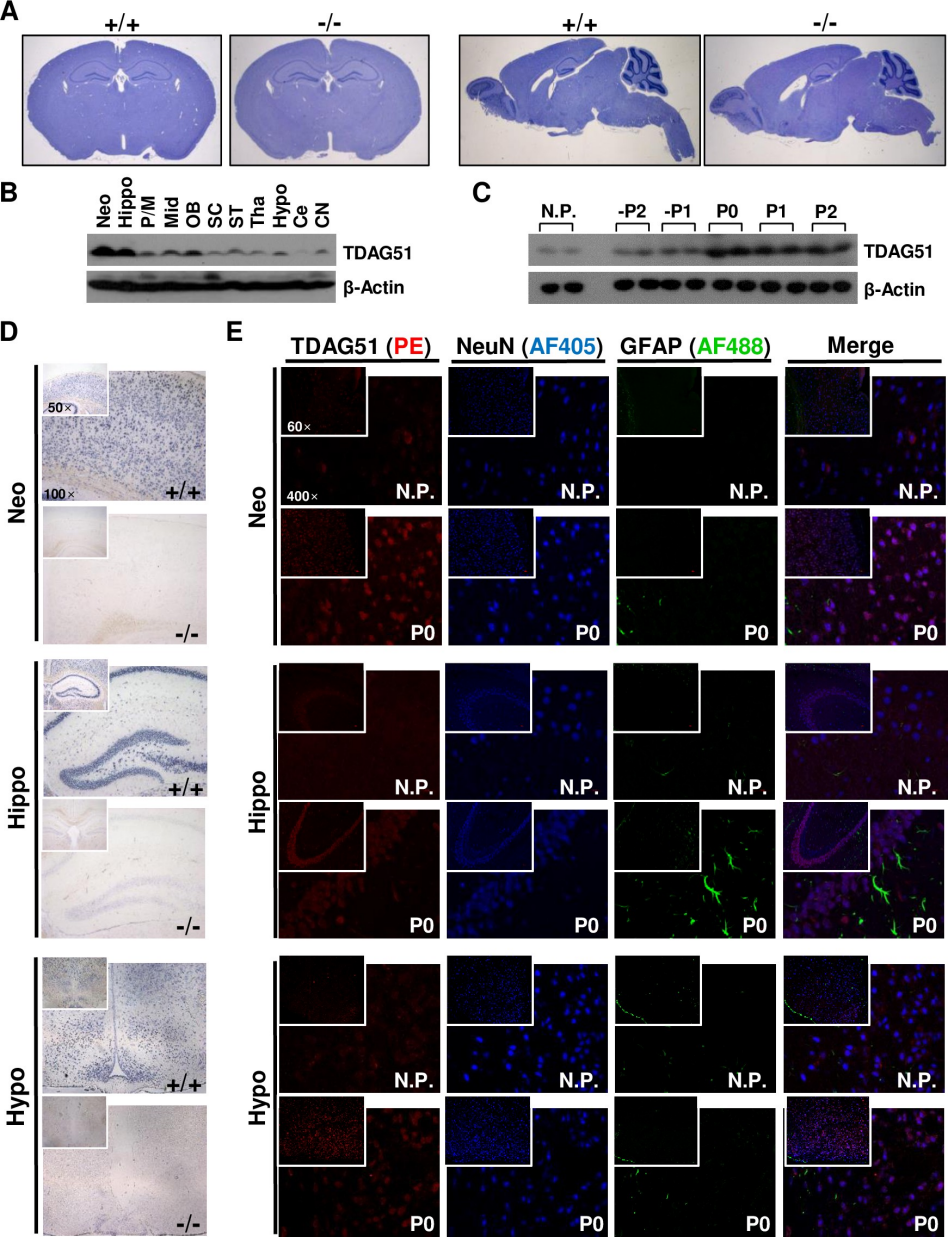
Expression of TDAG51 in brain tissues. (A) TDAG51 expression in brain sections from nonpregnant female mice. Histological images of coronal brain sections (top and left panels) and sagittal brain sections (top and right panels) were obtained by hematoxylin staining. +/+, TDAG51^+/+^. -/-, TDAG51^-/-^. (B) TDAG51 expression in mouse brain tissues. TDAG51 expression in brain tissues from nonpregnant female mice was analyzed by a western blot analysis with an anti-TDAG51 antibody. β-Actin was used as a loading control. Neo, neocortex. Hippo, hippocampus. P/M, pons/medulla. Mid, midbrain. OB, olfactory bulb. SC, spinal cord. ST, stria terminalis. Tha, thalamus. Hypo, hypothalamus. Ce, cerebellum. CN, cerebral nuclei. (C) Comparison of the TDAG51 expression levels in the brain during pregnancy, parturition and postpartum periods. TDAG51 expression levels in two mice per group were analyzed by a western blot analysis using an anti-TDAG51 antibody. Nonpregnant female mice (N.P.) were used as controls. (D) *In situ* hybridization analysis of TDAG51 expression in mouse brain tissues. TDAG51 expression was analyzed by an *in situ* hybridization analysis using DIG-labeled RNA probes on P0. All images were photographed at a 50× or 100× magnification. (E) TDAG51 expression in neuronal cells in mouse brain tissues. Mouse brain tissues were stained with an anti-TDAG51 PE-conjugated, anti-GFAP Alexa Fluor 488 (AF488)-conjugated and anti-NeuN Alexa Fluor 405 (AF405)-conjugated antibodies. All images were photographed at a 60× or 400× magnification. Images from the same observed field were merged.

Depressive disorders are closely linked to morphological and functional abnormalities in brain tissues [41]. However, we observed no gross morphological differences in the brain tissues between the TDAG51^+/+^ and TDAG51^-/-^ dams (Fig 3A). Thus, we examined the expression of TDAG51 in the brain tissues. TDAG51 was abundantly expressed in most brain tissues, and high levels were observed in the neocortex and hippocampus relative to the other areas (Fig 3B). Moreover, TDAG51 expression in the pregnant mice was higher during the prenatal, parturition and postnatal days from -P2 to P2 compared to that in the nonpregnant female mice (Fig 3C). Similar to the results shown in Fig 3B, we observed higher TDAG51 expression in the neocortex and hippocampus relative to that in the hypothalamus by *in situ* hybridization analysis (Fig 3D). To examine the cell types expressing TDAG51 in the brain tissues, the brain tissues were stained with an anti-TDAG51 immunofluorescence antibody, a marker of neuronal cells (anti-NeuN antibody) and a marker of astrocytes and neoplastic cells of glial origin (anti-glial fibrillary acidic protein (GFAP) antibody). We observed that the enhanced TDAG51 expression in the neocortex, hippocampus and hypothalamus of the postpartum brain tissues (P0) compared to that in the nonpregnant female mice was mainly detected in neuronal cells (Fig 3E). Taken together, these results indicate that the enhanced expression of TDAG51 in brain tissues during late pregnancy and the early postpartum period might be linked to the regulation of depressive-like behavior.

### Effects of brain-specific expression of the TDAG51 transgene in TDAG51^-/-^ dams

**Fig 4:**
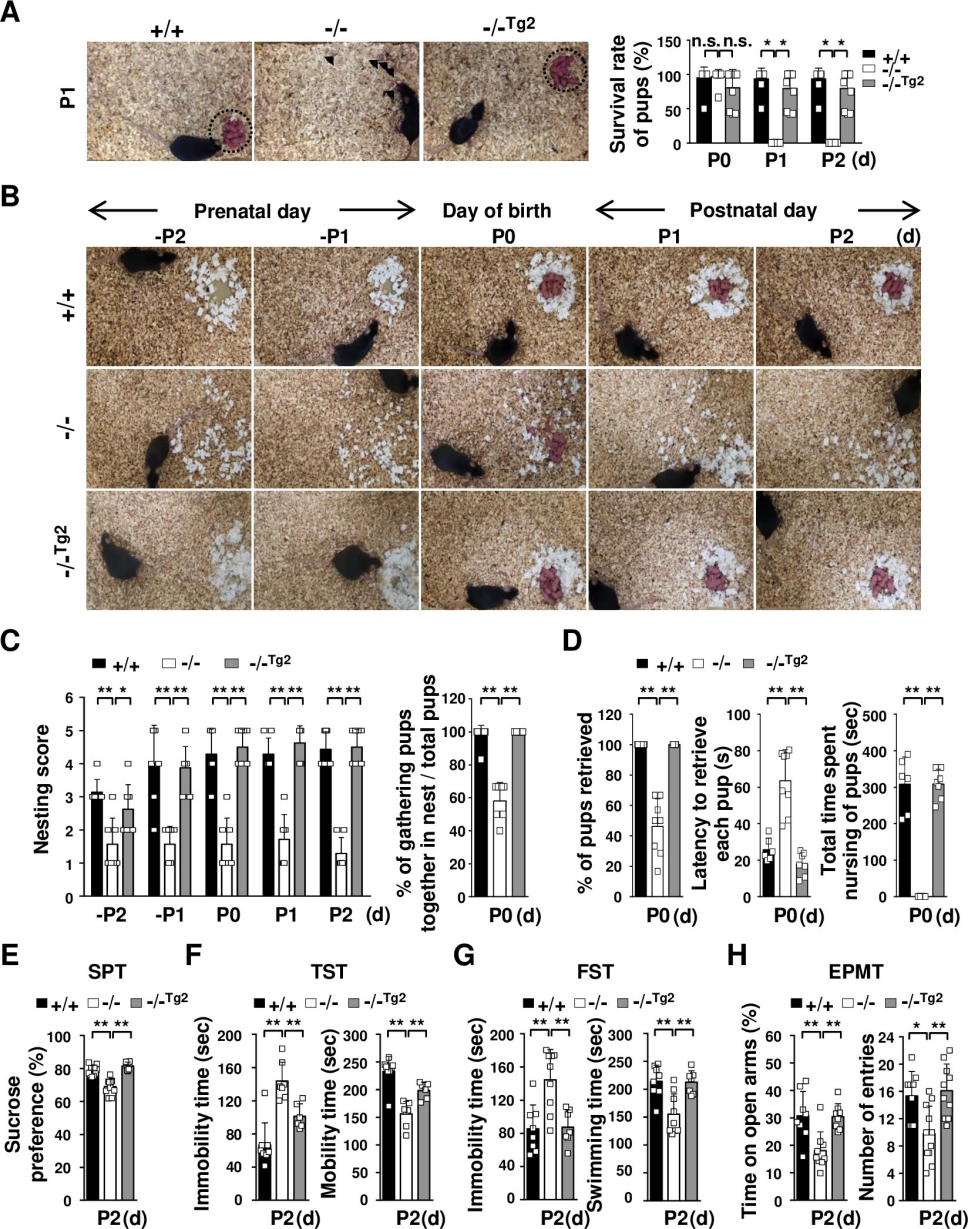
Effects of the brain-specific expression of the TDAG51 transgene in TDAG51^-/-^ dams. (A) Rescue effect (as observed in the pup survival test) of brain-specific TDAG51 transgene expression in TDAG51^-/-^ dams. Transgenic lines 2 (TDAG51^-/-Tg2^) and 3 (TDAG51^-/-Tg3^) expressing the TDAG51 transgene in the brain of TDAG51^-/-^ female mice were generated. The photographs were obtained on P1 (left panel). Arrowheads indicate dead pups. Dotted circles indicate pups gathered in a nest. Pup survival rate was measured from P0 to P2 (right panel). Black bar (+/+), TDAG51^+/+^ dams. White bar (-/-), TDAG51^-/-^ dams. Gray bar (-/-^Tg2^), TDAG51^-/-Tg2^ dams. (B) Rescue effect on nest building behavior in TDAG51^-/-Tg2^ pregnant mice. Photographs of the nest were obtained from -P2 to P2. (C) Measurement of nest building abilities. Nesting scores of TDAG51^+/+^, TDAG51^-/-^ and TDAG51^-/-Tg2^ pregnant mice were recorded (left panel). The number of pups gathered in a nest expressed as a percentage of the total number of neonatal pups measured on P0 (right panel). (D) Rescue effect on pup retrieval behavior in TDAG51^-/-Tg2^ dams. Left panel, percentage of retrieved pups per dam. Middle panel, latency to retrieve each pup by TDAG51^-/-^ dams. Right panel, impaired nursing behavior in TDAG51^-/-^ dams. (E) SPT. (F) TST. (G) FST. (H) EPMT. *p<0.05. **p<0.01. n.s., not significant.

To analyze whether TDAG51 deficiency in brain tissue causes maternal care defects and depressive-like behavior during the early postpartum period, we generated two transgenic founder mice through microinjections of a transgenic vector expressing the TDAG51 transgene under the control of the brain-specific BAI1-AP4 promoter (S2A Fig), which is expressed mainly in the cerebral cortex and hippocampus, as previously described [42]. We obtained two lines (TDAG51^-/-Tg2^ and TDAG51^-/-Tg3^) of transgenic mice by crossing the founder mice with TDAG51^-/-^ mice (S2B Fig). The transgene expression levels in the brain tissues of the TDAG51^-/-Tg2^ and TDAG51^-/-Tg3^ transgenic mice were analyzed by RT-PCR and immunofluorescence analyses (S2C and S2D Fig). We observed that the resulting pups were normally fostered by the TDAG51^-/-Tg2^ dams (Fig 4A (left)). Moreover, the survival rate of the pups that were born to the TDAG51^-/-Tg2^ dams was restored to that observed among the TDAG51^+/+^ dams (Fig 4A (right)). In the maternal behavior tests, the TDAG51^-/-Tg2^ dams showed dramatically rescued maternal behavior compared to the TDAG51^-/-^ dams (Fig 4B–4D). Subsequently, we further evaluated the depressive-like phenotypes of the TDAG51^-/-Tg2^ dams. The TDAG51^-/-Tg2^ dams exhibited SPT, TST and FST values that were restored from the levels observed in the TDAG51^-/-^ dams to those observed in the TDAG51^+/+^ dams (*p*<0.01) (Fig 4E–4G). In the EPMT, the reduced time spent in the open arms and the decreased numbers of entries into the open arms observed in the TDAG51^-/-^ dams were also restored in the TDAG51^-/-Tg2^ dams (*p*<0.01) (Fig 4H). Corresponding to the results shown in Fig 4, we observed that the transgenic TDAG51^-/-Tg3^ dams had similar phenotypes restored to those observed in the TDAG51^-/-Tg2^ dams (S3 Fig). Taken together, these results indicate that the abnormal maternal behavior and enhanced susceptibility to depressive-like behavior in the TDAG51^-/-^ dams can be restored by the brain-specific expression of TDAG51.

### Neuroendocrine dysregulation in TDAG51^-/-^ dams after parturition

**Fig 5:**
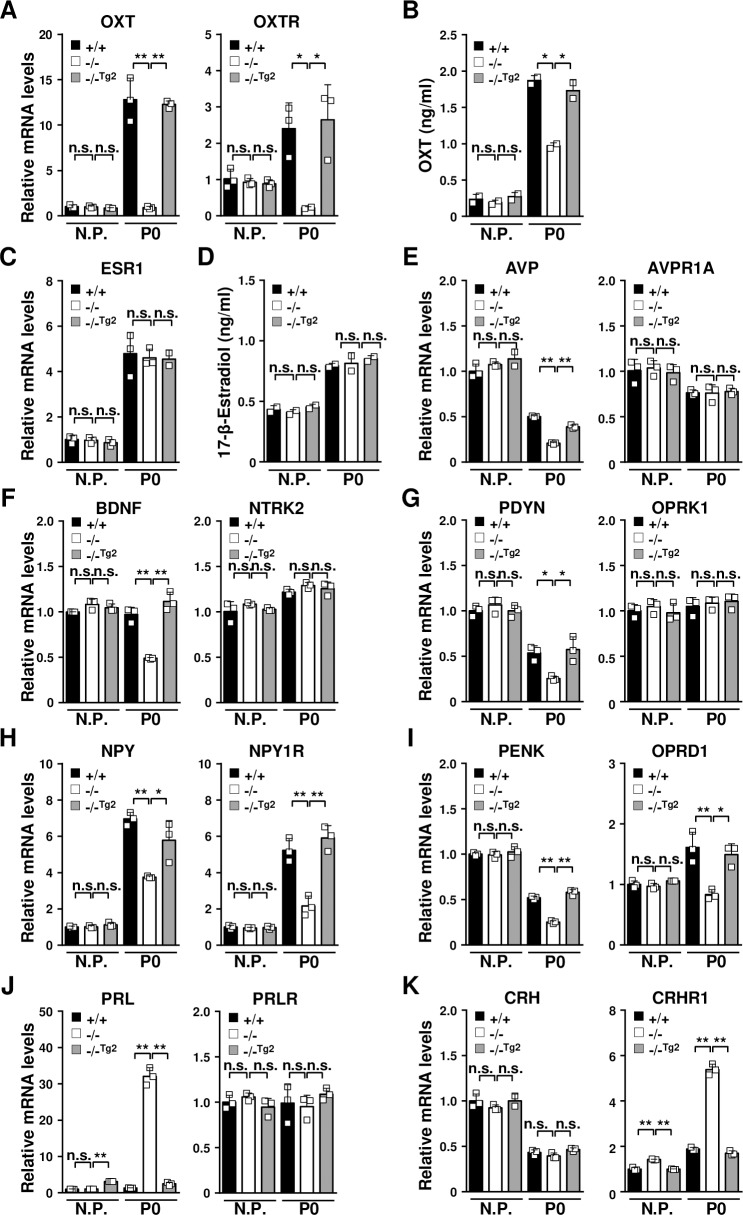
Dysregulation of neuroendocrine factors in the brain tissue of TDAG51^-/-^ dams after parturition. (A) Impaired expression of OXT and its receptor OXTR in TDAG51^-/-^ dams after parturition. Total RNA isolated from brain tissues was analyzed by real-time PCR using specific primers. N.P., nonpregnant mice. Black bar (+/+), TDAG51^+/+^ dams. White bar (-/-), TDAG51^-/-^ dams. Gray bar (-/-^Tg2^), TDAG51^-/-Tg2^ dams. (B) Serum OXT levels in TDAG51^-/-^ dams after parturition. Serum OXT levels were analyzed using ELISA. (C) No difference in ESR1 expression was observed in the TDAG51^-/-^ dams. (D) No difference in the serum estrogen levels was observed in the TDAG51^-/-^ dams. Serum estrogen (17-β-estradiol) levels after parturition were analyzed using ELISA. (E) Downregulation of AVP expression in TDAG51^-/-^ dams after parturition. (F) Downregulation of BDNF expression in TDAG51^-/-^ dams after parturition. (G) Downregulation of PDYN expression in TDAG51^-/-^ dams after parturition. (H) Downregulation of NPY and its receptor NPY1R expression in TDAG51^-/-^ dams after parturition. (I) Downregulation of PENK and its receptor OPRD1 expression in TDAG51^-/-^ dams after parturition. (J) Upregulation of PRL expression in TDAG51^-/-^ dams after parturition. (K) Upregulation of CRHR1 expression in TDAG51^-/-^ dams after parturition. *p<0.05. **p<0.01. n.s., not significant.

Neuroendocrine dysregulation is closely associated with postpartum depression, anxiety and maternal behavior [1, 2, 43]. Research has shown that the neuropeptide OXT and the female hormone estrogen are implicated in maternal behavior and postpartum depression [35, 44, 45]. Thus, we examined the expression levels of OXT, OXTR and ESR1 in the brain tissues of TDAG51^+/+^, TDAG51^-/-^ and TDAG51^-/-Tg2^ dams. The levels of OXT and OXTR in the brain tissues of the TDAG51^-/-^ dams were significantly lower on P0 than those in the TDAG51^+/+^ and TDAG51^-/-Tg2^ dams (*p*<0.01), whereas the OXT and OXTR levels were not altered in the TDAG51^-/-^ nonpregnant female mice (Fig 5A). Consistent with these results, the levels of plasma OXT were significantly reduced in the TDAG51^-/-^ dams on P0 (*p*<0.01) (Fig 5B). However, the ESR1 expression levels in the brain tissues and the 17-β-estradiol levels in the plasma were not affected by the TDAG51 deficiency in either the nonpregnant or postpartum mice (Fig 5C and 5D). Then, we further explored the differential expression of other neuroendocrine factors in the brain tissues of the TDAG51^-/-^ dams (Fig 5E–5K). On P0, the expression levels of AVP, BDNF and prodynorphin (PDYN) in the brain tissues of the TDAG51^-/-^ dams were significantly lower than those in the TDAG51^+/+^ (*p*<0.05) and TDAG51^-/-Tg2^ dams (*p*<0.05), whereas there were no detectable changes in the expression levels of their receptors (Fig 5E–5G). Moreover, the levels of neuropeptide Y (NPY) and its receptor NPYR and the levels of proenkephalin (PENK) and its receptor opioid receptor δ1 (OPRD1) were significantly decreased (*p*<0.01) in the brain tissue of the TDAG51^-/-^ dams (Fig 5H and 5I). However, the PRL and CRHR1 expression levels were oppositely upregulated in the brain tissue of the TDAG51^-/-^ dams (*p*<0.01) (Fig 5J and 5K). Corresponding to the results shown in Fig 4, similar results were obtained in the brain tissue of the transgenic TDAG51^-/-Tg3^ dams (S4 Fig). Altogether, these results indicate that the expression of TDAG51 in the brain tissue after parturition is closely associated with the altered regulation of neuroendocrine factors and their receptors.

### Altered gene expression in the brain tissue of TDAG51^-/-^ dams after parturition

**Fig 6:**
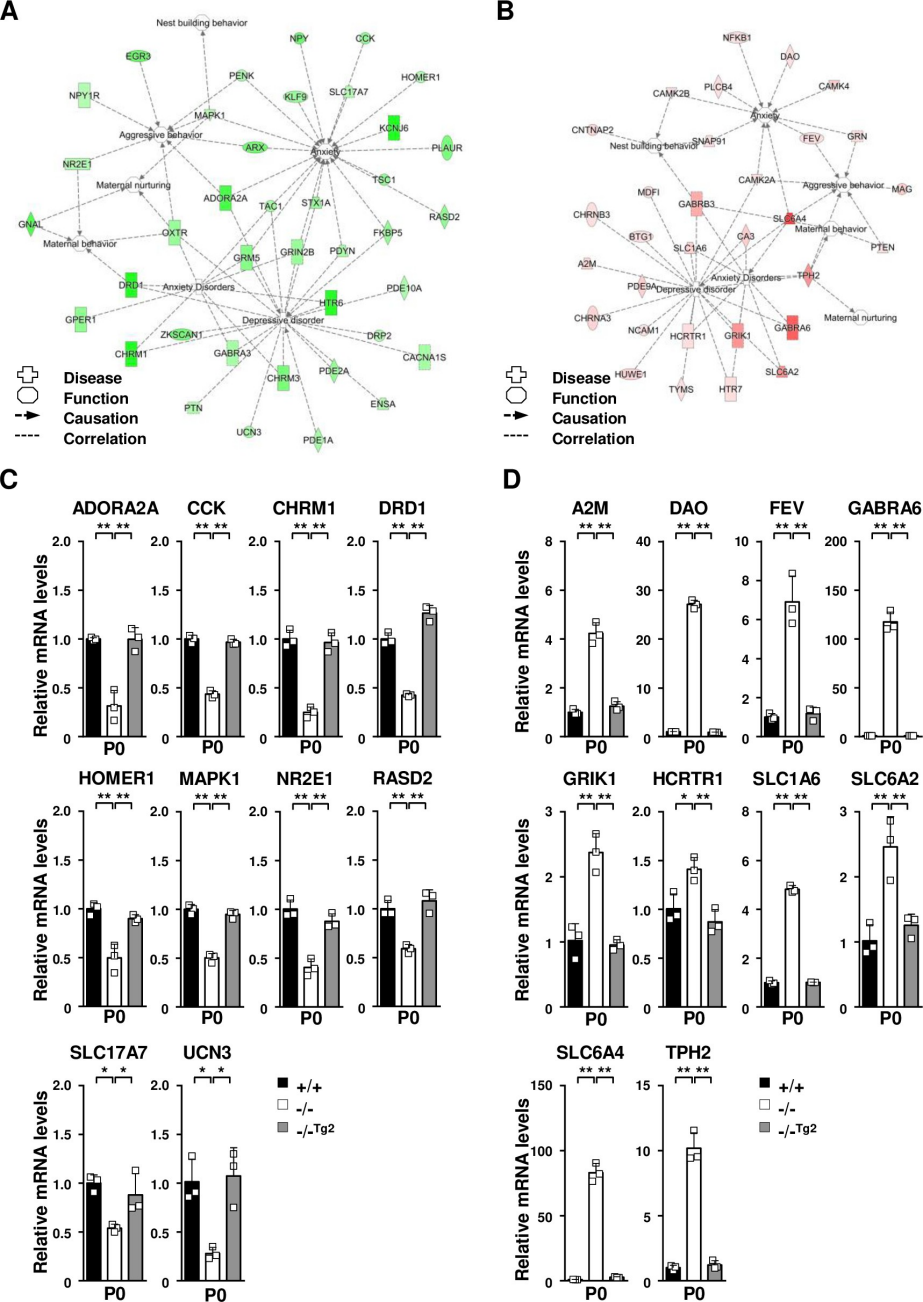
Altered gene expression in the brain tissue of TDAG51^-/-^ dams after parturition. (A) Prediction of the functional networks of the 39 downregulated genes listed in Table 1. Node color intensity indicates the degree of the downregulation of gene expression as follows: a greater intensity of green represents a higher degree of downregulation. Inverted triangle, kinase; dotted square, growth factor; vertical rectangle, G-protein coupled receptor; dotted vertical rectangle, ion channel; horizontal rectangle, ligand-dependent nuclear receptor; vertical diamond, enzyme; trapezoid, transporter; horizontal ellipse, transcription regulator; circle, other. (B) Prediction of the functional networks of the 31 upregulated genes listed in Table 2. Node color intensity indicates the degree of the upregulation of gene expression as follows: a greater intensity of red represents a higher degree of upregulation. (C) Real-time PCR analysis of downregulated genes selected from the list shown in Table 1. Total RNA isolated from brain tissues was analyzed using real-time PCR with specific primers. Black bar (+/+), TDAG51^+/+^ dams. White bar (-/-), TDAG51^-/-^ dams. Gray bar (-/-^Tg2^), TDAG51^-/-Tg2^ dams. (D) Real-time PCR analysis of upregulated genes selected from the list shown in Table 2. *p<0.05. **p<0.01.

To further investigate additional alterations in gene expression potentially affecting the depressive-like and abnormal maternal behaviors in the TDAG51^-/-^ dams, we conducted a microarray analysis using a Whole Mouse Genome Microarray kit (4×44k, 41,174 gene features) and compared the transcripts in the postpartum brain tissues between the TDAG51^-/-^ and TDAG51^+/+^ dams and between the TDAG51^-/-^ and TDAG51^-/-Tg2^ dams. We identified 2,374 differentially expressed genes (1,348 upregulated and 1,026 downregulated gene targets) showing a relative change of at least 2-fold (*p*<0.05) between the TDAG51^-/-^ postpartum brain tissues and TDAG51^+/+^ and TDAG51^-/-Tg2^ tissues (https://www.ncbi.nlm.nih.gov/geo/query/acc.cgi?acc=GSE118675). Subsequently, we analyzed these 2,374 genes using the IPA functional annotation clustering tool to examine their biological relationships according to Gene Ontology annotations. Among the genes categorized into 109 biologically categorized groups, 247 genes were categorized in the behavior and psychological disorder groups (the filter was set to *p*<0.05 after FDR correction). To further narrow the list of potential target genes, we selected 7 functional pathways of interest, including those related to aggressive behavior, maternal behavior, maternal nurturing, nest building behavior, anxiety, anxiety disorder and depressive disorder, from these two groups. In total, we obtained 70 potential target genes that were involved in 7 functional pathways (Fig 6A and 6B); of these genes, 39 were downregulated (Table 1), and 31 were upregulated (Table 2) in the TDAG51^-/-^ postpartum brain tissues compared to their levels in the TDAG51^+/+^ and TDAG51^-/-Tg2^ tissues.

**Table 1:** Downregulated genes in TDAG51^-/-^ brain tissues after parturition.

				Fold change	
Symbol	Gene ID	Protein name	Category	-/- vs. +/+	-/- vs. -/-^Tg2^
ADORA2A	11540	Adenosine A2a receptor	A, B, D	-4.9	-4.3
ARX	11878	Aristaless related homeobox	A, B	-4.0	-2.1
CACNA1S	12292	Calcium voltage-gated channel subunit alpha1 S	D	-2.3	-2.4
CCK	12424	Cholecystokinin	B	-3.1	-2.6
CHRM1	12669	Cholinergic receptor muscarinic 1	C, D	-6.4	-4.7
CHRM3	12671	Cholinergic receptor muscarinic 3	C, D	-3.5	-4.7
DRD1	13488	Dopamine receptor D1	C, D, E	-5.9	-5.8
DRP2	13497	Dystrophin related protein 2	D	-2.1	-2.8
EGR3	13655	Early growth response 3	A	-4.3	-5.5
ENSA	56205	Endosulfine alpha (alpha-endosulfine)	B	-2.2	-2.5
FKBP5	14229	FK506 binding protein 5	B, D	-2.9	-2.1
GABRA3	14396	Gamma-aminobutyric acid type A (GABA_A_) receptor alpha3 subunit	C, D	-2.3	-2.1
GNAL	14680	G protein subunit alpha L	E, F	-4.7	-2.4
GPER1	76854	G protein-coupled estrogen receptor 1	C	-3.0	-2.1
GRIN2B	14812	Glutamate ionotropic receptor NMDA type subunit 2B	B, C, D	-2.7	-2.7
GRM5	108071	Glutamate metabotropic receptor 5 (mGluR5)	B, C, D	-3.2	-2.6
HOMER1	26556	Homer scaffolding protein 1	B	-2.3	-2.4
HTR6	15565	5-Hydroxytryptamine (serotonin, 5-HT) receptor 6 (5-HT_6_ receptor)	C, D	-5.8	-8.8
KCNJ6	16522	Potassium voltage-gated channel subfamily J member 6	B	-22.9	-6.0
KLF9	16601	Kruppel like factor 9	B	-2.8	-2.0
MAPK1	26413	Mitogen-activated protein kinase 1	A, B, F, G	-2.2	-2.1
NPY	109648	Neuropeptide Y	B	-4.1	-5.3
NPY1R	18166	Neuropeptide Y receptor Y1	A	-2.0	-2.5
NR2E1	21907	Nuclear receptor subfamily 2 group E member 1	A, E	-2.1	-2.2
OXTR	18430	Oxytocin receptor	A, C, D, E, F	-2.7	-2.2
PDE10A	23984	Phosphodiesterase 10A	D	-2.3	-2.1
PDE1A	18573	Phosphodiesterase 1A	D	-2.4	-2.8
PDE2A	207728	Phosphodiesterase 2A	D	-2.8	-3.0
PDYN	18610	Prodynorphin	B, D	-2.5	-3.8
PENK	18619	Proenkephalin	A, B	-2.3	-2.5
PLAUR	18793	Plasminogen activator, urokinase receptor	B	-3.5	-3.6
PTN	19242	Pleiotrophin	D	-2.2	-2.4
RASD2	75141	RASD family member 2 (GTP-binding protein Rhes)	B	-3.0	-2.3
SLC17A7	72961	Solute carrier family 17 member 7 (Vesicular glutamate transporter 1)	B	-2.1	-2.7
STX1A	20907	Syntaxin 1A	B, D	-2.9	-2.6
TAC1	21333	Tachykinin precursor 1	B, C, D	-2.7	-3.1
TSC1	64930	TSC complex subunit 1 (Hamartin)	B	-3.0	-5.2
UCN3	83428	Urocortin 3	D	-2.4	-5.9
ZKSCAN1	74570	Zinc finger with KRAB and SCAN domains 1	D	-2.4	-5.9

Downregulated genes in TDAG51^-/-^ vs. TDAG51^+/+^ or TDAG51^-/-^ vs. TDAG51^-/-Tg2^ brain tissues (P0) after parturition. Genes differentially expressed between TDAG51^-/-^ and TDAG51^+/+^ control or TDAG51^-/-Tg2^ brain tissues were categorized by an IPA analysis with the following disease and function annotations: A, Aggressive behavior; B, Anxiety; C, Anxiety disorder; D, Depressive disorder; E, Maternal behavior; F, Maternal nurturing; and G, Nest building behavior. Gene ID, Gene identification number.

**Table 2:** Upregulated genes in TDAG51^-/-^ brain tissues after parturition.

				Fold change	
Symbol	Gene ID	Protein name	Description	-/- vs. +/+	-/- vs. -/-^Tg2^
A2M	232345	Alpha-2-macroglobulin	D	3.9	5.2
BTG1	12226	BTG anti-proliferation factor 1	D	2.3	2.1
CA3	12350	Carbonic anhydrase 3	C, D	4.6	13.5
CAMK2A	12322	Calcium/calmodulin dependent protein kinase II alpha	A, B, D	2.2	2.2
CAMK2B	12323	Calcium/calmodulin dependent protein kinase II beta	B, G	3.3	3.4
CAMK4	12326	Calcium/calmodulin dependent protein kinase IV	D	4.7	3.1
CHRNA3	110834	Cholinergic receptor nicotinic alpha 3 subunit	D	3.9	2.0
CHRNB3	108043	Cholinergic receptor nicotinic beta 3 subunit	G	2.2	2.0
CNTNAP2	66797	Contactin associated protein-like 2	B	3.9	3.1
DAO	13142	D-amino acid oxidase	B	2.2	2.1
FEV	260298	ETS transcription factor	A, B	2.2	7.3
GABRA6	14399	Gamma-aminobutyric acid type A receptor alpha6 subunit	C, D	15.2	15.5
GABRB3	14402	Gamma-aminobutyric acid type A receptor beta3 subunit	C, D, E, G	7.8	4.0
GRIK1	14805	Glutamate ionotropic receptor kainate type subunit 1	C, D	10.0	2.7
GRN	14824	Granulin precursor	A, B	2.1	3.0
HCRTR1	230777	Hypocretin receptor 1	C, D	2.2	2.3
HTR7	15566	5-Hydroxytryptamine (serotonin, 5-HT) receptor 7 (5-HT_7_ receptor)	C, D	2.6	6.4
HUWE1	59026	HECT, UBA and WWE domain containing 1	D	4.2	3.0
MAG	17136	Myelin associated glycoprotein	A	4.5	5.3
MDFI	17240	MyoD family inhibitor	D	2.4	3.1
NCAM1	17967	Neural cell adhesion molecule 1	D	2.1	2.5
NFKB1	18033	Nuclear factor kappa B subunit 1 (NFκB p105 subunit)	B	2.9	9.2
PDE9A	18585	Phosphodiesterase 9A	D	4.4	2.7
PLCB4	18798	Phospholipase C beta 4	B	3.0	2.3
PTEN	320193	Phosphatase and tensin homolog	A, E	2.1	2.3
SLC1A6	20513	Solute carrier family 1 member 6 (high affinity Asp/Glu transporter)	C, D	4.3	4.3
SLC6A2	20538	Solute carrier family 6 member 2 (norepinephrine transporter)	C, D	10.8	11.8
SLC6A4	15567	Solute carrier family 6 member 4 (serotonin transporter)	A, B, C, D	19.6	11.8
SNAP91	20616	Synaptosome associated protein 91	B, G	2.4	3.4
TPH2	216343	Tryptophan hydroxylase 2	A, C, D, E, F	10.3	2.8
TYMS	22171	Thymidylate synthetase	D	2.9	2.6

Upregulated genes in TDAG51^-/-^ vs. TDAG51^+/+^ or TDAG51^-/-^ vs. TDAG51^-/-Tg2^ brain tissues (P0) after parturition. Genes differentially expressed between TDAG51^-/-^ and TDAG51^+/+^ control or TDAG51^-/-Tg2^ brain tissues were categorized by an IPA analysis with the following disease and function annotations: A, Aggressive behavior; B, Anxiety; C, Anxiety disorder; D, Depressive disorder; E, Maternal behavior; F, Maternal nurturing; and G, Nest building behavior. Gene ID, Gene identification number.

To further confirm the data obtained by the microarray and IPA analyses, we analyzed the changes in the expression levels of genes selected from Table 1 or Table 2 by quantitative real-time PCR analysis. Of the downregulated genes shown in Table 1, the expression levels of adenosine A2a receptor (ADORA2A), cholecystokinin (CCK), cholinergic receptor muscarinic 1 (CHRM1), dopamine receptor D1 (DRD1), homer scaffolding protein 1 (HOMER1), MAPK1, nuclear receptor subfamily 3 group E member 1 (NR2E1), RASD family member 2 (RASD2), solute carrier family 17 member 7 (SLC17A7) and urocortin 3 (UCN3) in the brain tissues of the TDAG51^-/-^ dams were significantly lower (*p*<0.05 or *p*<0.01) than those in the TDAG51^+/+^ or TDAG51^-/-Tg2^ dams (Fig 6C). However, of the upregulated genes shown in Table 2, the expression levels of α2-macroglobulin (A2M), D-amino acid oxidase (DAO), ETS transcription factor Fev (FEV), GABA type A receptor α6 subunit (GABRA6), glutamate ionotropic receptor kainate type subunit 1 (GRIK1), hypocretin receptor 1 (HCRTR1), SLC1A6, SLC6A2, SLC6A4 and TPH2 in the brain tissues of the TDAG51^-/-^ dams were significantly higher (*p*<0.05 or *p*<0.01) than those in the TDAG51^+/+^ and TDAG51^-/-Tg2^ dams (Fig 6D). Corresponding to the results shown in Fig 6C and 6D, similar results were obtained in the brain tissue of the transgenic TDAG51^-/-Tg3^ dams (S5 Fig). Altogether, these results indicate that TDAG51 acts as a global regulator by promoting or repressing the expression of regulatory pathways involved in maternal behavior, anxiety and depression.

## Discussion

Many studies elucidating the role of TDAG51 have been published; however, these studies have only examined its roles in the regulation of cell growth/differentiation, cell survival/death and tumorigenesis [46]. In our present study, we provide new insight into the function of TDAG51 in the development of depressive-like and abnormal maternal behavior. Our results show that TDAG51 deficiency is closely associated with reduced maternal care and enhanced susceptibility to depressive-like behavior after parturition. Interestingly, most phenotypic features are similar to and overlap with the characteristics of postpartum psychiatric illness in human patients [2, 4]. Postpartum psychiatric disorder, which is also known as postpartum depression, is a severe emotional and mental disease that can affect women typically after parturition [47]. Postpartum blues or baby blues represent the mildest type, resulting in a depressed mood experienced shortly after parturition. However, postpartum depression and postpartum psychosis have clinically significant symptoms, including severely depressed mood, insomnia, anhedonia, anxiety, self-injury, and increased risks of suicide, infant abuse and infanticide [48]. Thus, the phenotype of the TDAG51^-/-^ dams exhibiting maternal care defects and anxiety-like and depressive-like behaviors after parturition is more closely related to postpartum depression and postpartum psychosis than postpartum blues.

Failure to regulate neuroendocrine factors, including neurotrophic factors, neuroendocrine hormones and neurotransmitters, is closely related to the development of postpartum depression and abnormal maternal behavior [1, 2, 4, 28, 29]. OXT, which was originally known to stimulate labor and milk ejection during female reproduction, is a neuropeptide hormone that plays roles in maternal care, social recognition, stress regulation, mood and anxiety [49, 50]. Although the precise mechanisms of OXT’s effects in HPA modulation are not well defined, OXT has clinically been shown to facilitate improved social communication, reduced anxiety and anti-depressive effects [51]. Furthermore, the reduced level of OXT observed during pregnancy and parturition is closely associated with the development of postpartum depression [52]. Almost concurrent with OXT, the neuropeptide AVP has been implicated in the regulation of maternal behavior [53]. In particular, studies investigating the role of brain AVP in maternal behavior have focused more on its role in maternal aggression in defeating intruders [1]. The neuropeptide BDNF is critical for brain neurogenesis, including neuron survival, axon growth and synaptic plasticity [4]. Interestingly, the loss of BDNF in mice is associated with brain monoamine deficiencies and an increased appearance of stress-induced depressive-like behavior [4, 54]. In human patients, the reduced levels of BDNF in response to proinflammatory cytokines, psychological stress and cortisol stimulation have been reported to contribute to the development of depression [4, 55]. Furthermore, the loss of neuropeptides, such as NPY, PDYN and PENK, has also been associated with enhanced susceptibility to anxiety-like and depressive-like behavior in mice [56–58]. Interestingly and coincidently with these previous reports, we show that the gene expression levels of certain neuroendocrine factors, such as OXT, AVP, BDNF, PDYN, NPY and PENK, were significantly reduced in the postpartum brain tissues of the TDAG51^-/-^ dams (Fig 5). Many lines of evidence suggest that PRL plays a protective role in anxiety-like, depressive-like and maternal behavior, although the precise role of PRL remains controversial [33, 59–61]. According to the Allen Brain Atlas (http://mouse.brain-map.org/experiment/show/75861792) and other reports [62, 63], there is no significant expression of the PRL gene in the mouse brain, whereas PRLR expression is detected throughout the brain. There is also evidence suggesting that brain PRL is induced or acts under certain specific conditions, such as brain development and in response to brain injury or local inflammation [62]. Interestingly, we showed that PRL expression in the brain tissues of the TDAG51^-/-^ dams was upregulated at relatively high levels compared that in the TDAG51^+/+^ dams (Fig 5J). However, thus far, our data do not support the hypothesis that the relative PRL expression in the brain of the TDAG51^-/-^ dams is correlated to the physiologic level of brain PRL because we did not directly examine the levels of brain PRL in the cerebrospinal fluids or brain tissues of the TDAG51^-/-^ dams in our current study. Thus, our interest focuses on how the enhanced PRL levels in the brain tissues are involved in the behavioral defects in the TDAG51^-/-^ dams and how PRL expression is regulated in the brain tissues by TDAG51 deficiency. Further studies are required to elucidate these ideas. In our microarray analysis, we further identified that the gene expression levels of the monoamine neurotransmitter receptor, transporter and regulator significantly differed in the postpartum brain tissues between the TDAG51^-/-^ dams and the controls (Fig 6, Table 1 and Table 2). Interestingly, decreased expression levels of ADORA2A, CHRM1, DRD1, and SLC17A7 and increased expression levels of DAO, GABRA6, GRIK1, SLC1A6, SLC6A2, SLC6A4 and TPH2 were observed (Fig 6 and S5 Fig). Many genetic and clinical studies strongly support the role of monoamine neurotransmitters in maternal behavior [1]. Furthermore, depression is closely associated with low levels of monoamine neurotransmitters, particularly dopamine, serotonin, epinephrine and norepinephrine [29]. Thus, we hypothesize that TDAG51 expression induced in brain tissue by pregnancy and parturition stress is a crucial regulator controlling the levels of neuroendocrine factors and monoamine neurotransmitters that may regulate the development of abnormal maternal behavior and postpartum depression.

TDAG51 is considered a putative transcriptional regulator and has an N-terminal PHL domain and C-terminal PQ- and PH-repeat domains, but no DNA-binding domain [46]. The PHL domain, which is evolutionarily conserved, is required for various cellular processes, including cell survival, differentiation and tumor progression [17, 21, 46]. The C-terminal region of TDAG51, which contains both a PQ- and a PH-repeat domain, is considered a putative transcriptional activator [17, 46]. Human and mouse TDAG51 share an 89% amino acid sequence identity and have conserved PHL, PQ-repeat and PH-repeat domains [17]. However, in the GenBank database, TDAG51 orthologs were found only in vertebrates, including a wide range of mammalian species, and were not found in plants, invertebrates, lower eukaryotes or bacteria (S6 Fig). Interestingly, based on a comparative sequence analysis of TDAG51 orthologs in vertebrates, we observed that the PQ- and PH-repeat domains were found only in mammalian species, such as humans, chimpanzees, cows and rodents, and were not found in other vertebrates, such as zebrafish and frogs (S6 Fig). Thus, it is possible that the PQ- and PH-repeat domains in TDAG51 may play a particularly crucial role in regulating transcription in mammalian species but not nonmammalian vertebrates. Considering the potential for transcriptional regulation in mammalian species, we postulate that TDAG51 is a crucial regulator of the levels of neuroendocrine factors and monoamine neurotransmitters in mammalian brain tissues, which may explain the mechanism by which TDAG51 affects maternal behavior and postpartum depression.

In conclusion, in this study, we discovered a novel function of TDAG51 in the regulation of maternal behavior and postpartum depression and demonstrated that TDAG51 deficiency induces depressive-like and abnormal maternal behaviors after parturition. Our results also show that the loss of TDAG51 in postpartum brain tissues induces changes in the expression levels of various maternal, anxiety-like and depressive-like behavior-associated genes that regulate the levels of neuroendocrine factors and monoamine neurotransmitters. Thus, these findings suggest that TDAG51 acts as a maternal care-associated gene that may be involved in the development of abnormal maternal behavior and postpartum depression.

## Materials and methods

### Ethics statement

All animal care and use procedures were conducted in accordance with the guidelines of The Ethics Training Guidelines for Experiments on Animals of CNU Animal Research Center. All animal experiments were approved by the Animal Experiment Ethics Committee of Chungnam National University (approval No. CNU-00114, 00584 and 01025).

### Mice

The C57BL/6J mice were obtained from the Laboratory Animal Resource Center in KRIBB (Ochang, Korea). The TDAG51^-/-^ mice were produced as previously described [18]. The mice were housed 5 per cage in a room maintained at 22±3°C under a 12 h light-dark cycle (lights on at 8:00 a.m.) with ad libitum access to food and water. All behavioral tests were performed between 9:00 a.m. and 6:00 p.m. using 8- to 12-week-old mice maintained in a pathogen-free facility at Chungnam National University (Daejeon, Korea), and the number of mice used is shown in S2 Table. The mice were allowed to adept to the testing environment for at least 1 h prior to the start of the behavioral tests. To generate transgenic mice with the brain-specific expression of TDAG51 (TDAG51^Tg^), the murine *TDAG51* gene was cloned into a pBAI1-AP4 vector harboring the brain-specific promoter of the *angiogenesis inhibitor 1-associated protein 4* gene as previously described [42]. The TDAG51^Tg^ mice were generated by the transgenic facility of KAIST (Daejeon, Korea). The TDAG51^Tg^ lines were identified by PCR genotyping using the following primers: T51tg-F, 5’-ATG CTG GAG AAC AGC GGC TGC-3’ and T51tg-R: 5’- GGT ATG GCT GAT TAT GAT C-3’. The TDAG51^Tg^ lines were backcrossed to C57BL/6J mice for at least 5 generations. To obtain TDAG51Tg mice on a TDAG51^-/-^ genetic background (TDAG51-/-Tg), TDAG51Tg female mice were crossed with TDAG51^-/-^ male mice.

### Pup survival

Female mice showing vaginal plugs were individually separated. The survival rate of the newborn pups was recorded from parturition day (P0) to postnatal day 2 (P2). A pup cross-fostering test was performed as previously described [64]. Briefly, pregnant female mice were individually housed in separate cages. On P0, the pups born to the TDAG51^+/+^ and TDAG51^-/-^ dams were separated from their dams, wiped clean with water-soaked cotton tissues, and dipped in litter containing the urine and feces of their surrogate dam. The newborn pups born to the TDAG51^+/+^ dams or TDAG51^-/-^ dams were immediately exchanged to TDAG51^-/-^ surrogate dams or TDAG51^+/+^ surrogate dams, respectively, on P0. The survival rate of the pups placed with surrogate dams was measured on P2. To test the effect of the male mating partners on the pup survival, the survival rate of the pups born to the TDAG51^-/-^ dams in the presence of TDAG51^-/-^ or TDAG51^+/+^ male mating partners in foster cages was analyzed from P0 to P2.

### Maternal behavior tests

A nesting behavior test was performed as previously described [65]. Briefly, pregnant female mice were individually separated from their male mating partners. The mice were given nesting material made from cotton fibers on prenatal day 3 (-P3). Photographs of the nest building were taken from -P2 to P2. Then, the nesting scores (0–5) were measured according to the extent of the nest based on nesting standards (5, very well-nested; 4, well-nested; 3, nested; 2, slightly scattered; 1, scattered; or 0, no nest). The percentage of pups gathered in the nest among the total newborn pups was measured on P0. Pup retrieval was analyzed as previously described [66]. Briefly, each dam was left alone for 5 min in the home cage prior to the pup retrieval test, and then, the newborn pups were placed in the home cage on the opposite side from the nest. The number of pups retrieved to the nesting zone, the latency to retrieve each pup and the dam’s nursing time were measured by video-monitoring for 10 min.

### Depressive- and anxiety-like behavior tests

The SPT was performed as previously described [64, 67]. Briefly, each pregnant mouse was housed individually in the home cage on -P3. After parturition (P0), the pups were removed from the home cages, and the dams were adapted to a 1% sucrose solution for 24 h. Then, the positions of the water and 1% sucrose bottles were changed before the test on P1, and the consumption of water or 1% sucrose solution was measured for 24 h. The sucrose preference of the nonpregnant female mice was measured using the same methods. The results are expressed as percent intake (sucrose intake (g)/total liquid intake (g) x 100). After the SPT, the behavior tests were conducted on P2 in sequential order, i.e., EPMT, TST and FST, with a time interval for 4 h or 6 h for each behavioral test to minimize stress induced by the previous behavioral test as previously described [38, 39]. The EPMT was performed as previously described [66]. Briefly, each female mouse was placed in the center area of the maze with its head directed toward an open arm and allowed to move freely throughout the maze for 10 min; their behavior was video-recorded. The number of entries into the open arm and the percent of time spent in the open arm were analyzed. After the EPMT, the mice were allowed to rest for 4 h in their home cages before the following behavioral test. The TST was performed as previously described [67, 68]. Briefly, each female mouse was individually suspended by taping the tail to a vertical bar 15 cm above the floor for 5 min, and the behavior was video-recorded. All behavioral experiment records were analyzed blindly. After the TST, the mice were allowed to rest for 6 h in their home cages before the following behavioral test. The FST was performed as previously described [64, 67]. Briefly, the female mice were individually placed in an acrylic round transparent cylinder (29 cm diameter) filled with 25±2°C water to a depth of 15 cm, and the behavior was video-recorded for 5 min. Immobility was defined as floating motionless or making only movements necessary to keep the head above water. After 5 min, the mice were placed in a cage with clean paper bedding and dried. The water was cleaned and replaced at the beginning of each trial.

### Real-time PCR and ELISA

The real-time PCR analysis was performed as previously described [69, 70]. Briefly, whole brains obtained from female mice on P0 were placed in TRIzol reagent (Invitrogen, Carlsbad, CA, USA) and lysed using a homogenizer (Daihan Scientific, Seoul, Korea) at 15,000 × *g* for 15 s on ice. The total RNA was obtained using a TRIzol kit according to the manufacturer’s instructions. To quantify gene expression, real-time PCR was used as previously described [71]. Reverse transcription was performed using 2 μg of total brain RNA and M-MLV reverse transcriptase (USB, Cleveland, OH, USA); then, the cDNAs were subjected to a real-time PCR analysis with the appropriate primers (S2 Table) and IQ SYBR Green Supermix (Bio-Rad, Hercules, CA, USA) using a CFX Connect Real-time PCR Detection System (Bio-Rad). β-Actin was used as an internal normalization control. The relative mRNA level was analyzed using the 2-ΔΔ threshold cycle (ΔΔCT) method as previously described [72]. Briefly, the ΔCT value was calculated by the following equation: (CT_(target gene)_—CT_(β-actin)_). The ΔΔCT value was calculated by the following equation: (ΔCT_(each sample)_—ΔCT_(nonpregnant wild-type female)_). An ELISA was performed as previously described [73]. Briefly, sera were collected from the mice, centrifuged, and subjected to ELISA. The serum levels of OXT and 17-β-estradiol were measured using an ELISA reader (Bio-Rad) at 405 nm using OXT and 17-β-estradiol assay kits (Enzo Life Science, Farmingdale, NY, USA) following the manufacturer’s instructions.

### Microarray and gene network analyses

To compare the transcripts in the postpartum brain tissues between the TDAG51^-/-^ dams and TDAG51^+/+^ dams and between the TDAG51^-/-^ dams and TDAG51^-/-Tg2^ dams, cDNAs derived from whole brain tissues obtained on P0 were tested against a Whole Mouse Genome 4×44k Microarray kit (Agilent Biotechnologies, Santa Clara, CA, USA) according to the manufacturer’s instructions. To define the differentially expressed genes, the significance cut-offs for the ratios of TDAG51^-/-^ vs. TDAG51^+/+^ and TDAG51^-/-^ vs. TDAG51^-/-Tg2^ genes were set at a 2.0-fold change as previously described [74]. To assess the biological functions and relationships among the differentially expressed genes, a data set of 2-fold differentially expressed genes was analyzed using the core analysis program of the Ingenuity Pathway Analysis (IPA) tool (Ingenuity System, Redwood City, CA, USA) as previously described [75]. Based on the results of the core analysis, possible candidate genes were obtained from the categorized genes in the behavior and psychological disorders groups (the filter was set to *p*<0.05 after FDR correction). To further analyze the gene functions and biological relationships, candidate genes were obtained from the subcategorized genes in the following 7 functional pathways: aggressive behavior, anxiety, anxiety disorder, depressive disorder, maternal behavior, maternal nurturing and nest building behavior. The selected candidate genes were analyzed by the gene network analysis program of the IPA tool. To validate the microarray expression profile, the gene expression levels of the possible candidate genes in the brain tissues were subsequently analyzed by real-time PCR.

### Immunocytochemistry

The histological analysis and immunohistochemistry were performed as previously described [76, 77]. Briefly, female mice were perfused through the left cardiac ventricle with PBS, followed by 10% formalin under anesthesia. The brains were embedded in paraffin prior to a 48-h postfixation in 10% formalin. The paraffin blocks were sectioned at 3 μm, mounted onto glass slides, and stained with hematoxylin. For the immunofluorescence analysis, immunofluorescence was performed as previously described [78]. The antigen-retrieved slides were incubated in blocking buffer for 1 h at 25°C and treated with an anti-TDAG51 phycoerythrin (PE)**-**conjugated antibody (Santa Cruz Biotechnology, Santa Cruz, CA, USA), an anti-glial fibrillary acidic protein (GFAP, a marker of astrocytes and neoplastic cells of glial origin) Alexa Fluor 488-conjugated antibody (Santa Cruz Biotechnology) and anti-NeuN (a neuron-specific nuclear protein) Alexa Fluor 405-conjugated antibody (Novus Biological, Littleton, CO, USA) at 1:100 dilutions in blocking buffer for 16 h at 4°C. Finally, the stained slides were analyzed under an Olympus BX61 microscope (Olympus, Tokyo, Japan).

### Western blot analysis

The total brain tissue protein was harvested as previously described [79]. The brain samples were homogenized in lysis buffer (25 mM Tris-HCl (pH 7.5), 150 mM NaCl, 1 mM EDTA, 1 mM NaF, 1 mM sodium orthovanadate, 1 mM phenylmethylsulfonyl fluoride, 5% glycerol and 0.5% Triton X-100) at 14,000 × *g* for 15 s on ice. Then, the lysates were separated by centrifugation at 18,000 × *g* for 10 min at 4°C. The supernatants were analyzed using 10% SDS-PAGE, transferred to polyvinylidene difluoride membranes and immunoblotted with anti-β-actin or anti-TDAG51 antibodies (Santa Cruz Biotechnology).

### *In situ* hybridization

*In situ* hybridization was performed as previously described [80]. To generate the RNA probe, cDNAs were subcloned into a pGEM-T vector (Promega, Madison, WI, USA). RNA probes labeled by digoxigenin (DIG) were prepared using a DIG RNA labeling kit (Roche, Mannheim, Germany) following the manufacturer’s instructions. The DIG-labeled RNA probes were diluted 1:100 with *in situ* hybridization buffer (Sigma, St. Louis, MO, USA), and the slides were incubated in a humidified chamber for 16 h at 60°C. The detection was conducted using a DIG nucleic acid detection kit (Roche) following the manufacturer’s instructions.

### Statistical analysis

All data are expressed as the means±standard errors of the mean (S.E.M.), and the statistical analysis was conducted using SPSS v.24.0 software (IBM Corp., Armonk, NY, USA). Statistical significance was analyzed using a two-tailed Student *t*-test, one-way ANOVA and two-way ANOVA with repeated measures, followed by a *post hoc* Fisher test. The effects were considered significant at *p*<0.05.

## Supporting information

S1 Fig Histology and anatomy of TDAG51^+/+^ and TDAG51^-/-^ mice. (A) No differences in mammary gland formation and luminal secretion were observed between the TDAG51^+/+^ and TDAG51^-/-^ dams. The morphology of the mammary papillae (top panel) and histology of the mammary glands (bottom panel) in the TDAG51^+/+^ dam and TDAG51^-/-^ dam on P1 are shown. The magnified regions are indicated by boxes. GL, glandular lumen. GE, glandular epithelium. Arrows, luminal secretion. The alveolar sizes were compared (right panel). +/+, TDAG51^+/+^. -/-, TDAG51^-/-^. n.s., not significant. (B) No suckling problems were observed in the neonate pups. The anatomy of the neonate pups is shown with magnified images. Dotted circles indicate suckled milk in the stomach.
(TIF)

S2 Fig Generation of brain-specific TDAG51 transgenic mice. (A) The construction of a brain-specific TDAG51 transgene expression vector. A transgenic vector harboring a brain-specific BAI1-AP4 promoter, the murine TDAG51 gene and an SV40 poly A signal is outlined (left). Nucleotide positions are numbered based on the transcriptional start site, and the putative binding site of the transcription factors is marked on the BAI1-AP4 promoter. Arrows indicate the positions of the genotyping PCR primers. (B) Genotyping of transgenic mice. The transgenic mice genotypes were confirmed by a PCR analysis. +/+, TDAG51^+/+^. -/-, TDAG51^-/-^. Ctl, control (TDAG51 transgenic vector). -/-^Tg2^, transgenic line (Tg-line) 2 on the TDAG51^-/-^ genetic background. -/-^Tg3^, transgenic mice line 3 on the TDAG51^-/-^ genetic background. (C) TDAG51 expression in the brain of Tg-line mice. TDAG51 expression in the brains of Tg-line mice was compared with that in the livers of Tg-line mice by a quantitative RT-PCR analysis. In the RT control reaction, no reverse transcriptase (RTase) was added. (D) TDAG51 expression was visualized in the brain tissues of the Tg-line mice by an immunofluorescence analysis. Mouse brain tissues were stained with anti-TDAG51 PE-conjugated, anti-GFAP Alexa Fluor 488 (AF488)-conjugated and anti-NeuN Alexa Fluor 405 (AF405)-conjugated antibodies. All images were photographed at a 60× or 400× magnification. Images observed in the same field were merged. Neo, neocortex. Hippo, hippocampus.
(TIF)

S3 Fig Effects of the brain-specific expression of the TDAG51 transgene on TDAG51^-/-^ dams. (A) Rescue effect (pup survival test) of brain-specific TDAG51 transgene expression in TDAG51^-/-^ dams. Transgenic line 3 (TDAG51^-/-Tg3^) mice were generated by expressing TDAG51 in the brain of TDAG51^-/-^ female mice (S3 Fig). The photographs were obtained on P1 (left panel). Arrowheads indicate dead pups. Dotted circles indicate pups gathered in a nest. Survival of pups born to TDAG51^+/+^, TDAG51^-/-^ and TDAG51^-/-Tg3^ dams was measured from P0 to P2 (right panel). Black bar (+/+), TDAG51^+/+^ dams. White bar (-/-), TDAG51^-/-^ dams. Gray bar (-/-^Tg3^), TDAG51^-/-Tg3^ dams. (B) Rescue effect (nest building behavior) observed in the TDAG51^-/-Tg3^ pregnant mice. The photographs of nest building were obtained from -P2 to P2. (C) Measurement of the nest building score. Nesting scores of TDAG51^+/+^, TDAG51^-/-^ and TDAG51^-/-Tg3^ pregnant mice (left panel) were analyzed. The number of pups gathered in a nest expressed as a percentage of the total number of neonate pups measured on P0 (right panel). (D) Rescue effect on pup retrieval behavior observed in the TDAG51^-/-Tg3^ dams. Left panel, the percentage of retrieved pups per dam. Middle panel, latency to retrieve each pup by TDAG51^-/-^ dams. Right panel, impaired nursing of pups by TDAG51^-/-^ dams. (E) SPT. (F) TST. (G) FST. (H) EPMT. *p<0.05. **p<0.01. n.s., not significant.
(TIF)

S4 Fig Dysregulation of neuroendocrine factors in the brain tissue of TDAG51^-/-^ dams after parturition. (A) Impaired expression of OXT and its receptor OXTR in TDAG51^-/-^ dams after parturition. Total RNA isolated from brain tissues was analyzed by real-time PCR using specific primers (Table S2). N.P., nonpregnant mice. Black bar (+/+), TDAG51^+/+^ dams. White bar (-/-), TDAG51^-/-^ dams. Gray bar (-/-^Tg3^), TDAG51^-/-Tg3^ dams. (B) Serum OXT levels in TDAG51^-/-^ dams after parturition. Serum OXT levels were analyzed using ELISA. (C) No differences in ESR1 expression were observed in the TDAG51^-/-^ dams after parturition. (D) No differences in serum estrogen levels were observed in the TDAG51^-/-^ dams after parturition. Serum estrogen (17-β-estradiol) levels were analyzed using ELISA. (E) Downregulation of AVP expression in the TDAG51^-/-^ dams after parturition. (F) Downregulation of BDNF expression in the TDAG51^-/-^ dams after parturition. (G) Downregulation of PDYN expression in the TDAG51^-/-^ dams after parturition. (H) Downregulation of the expression of NPY and its receptor NPY1R in the TDAG51^-/-^ dams after parturition. (I) Downregulation of the expression of PENK and its receptor OPRD1 in the TDAG51^-/-^ dams after parturition. (J) Upregulation of PRL expression in the TDAG51^-/-^ dams after parturition. (K) Upregulation of CRHR1 expression in the TDAG51^-/-^ dams after parturition. *p<0.05. **p<0.01. n.s., not significant.
(TIF)

S5 Fig Altered gene expression in the brain tissue of TDAG51^-/-^ dams after parturition. (A) Real-time PCR analysis of downregulated genes selected from the list shown in Table 1. Total RNA isolated from brain tissues was analyzed by real-time PCR using specific primers (Table S2). Black bar (+/+), TDAG51^+/+^ dams. White bar (-/-), TDAG51^-/-^ dams. Gray bar (-/-^Tg3^), TDAG51^-/-Tg3^ dams. (B) Real-time PCR analysis of upregulated genes selected from the list shown in Table 2. *p<0.05. **p<0.01.
(TIF)

S6 Fig Schematic structure of the TDAG51 domains and amino acid sequence analysis. (A) Schematic illustration of the murine TDAG51 domains. The PHL domain, PQ-repeat domain and PH-repeat domain are indicated along with the amino acid residues. PHL-N, N-terminal region of the PHL domain. PHL-C, C-terminal region of the PHL domain. (B) Amino acid sequence alignment of TDAG51 orthologs. Identical residues among the TDAG51 orthologs are shaded in black. Residues of the PHL, PQ and PH domains are marked by a red line. The sequences shown were obtained from the GenBank database under the following codes: hTDAG51 (*Homo sapiens*, BC110820), pTDAG51 (*Pan troglodytes*, XM_001161528), bTDAG51 (*Bos taurus*, BC134549), mTDAG51 (*Mus musculus*, U44088), rTDAG51 (*Rattus norvegicus*, AF192802), xTDAG51 (*Xenopus laevis*, NM_001097698) and dTDAG51 (*Danio rerio*, NM_001006011). (C) Phylogenetic analysis of the TDAG51 orthologs. The scale bar indicates the nucleotide substitutions per site.
(TIF)

S1 Table Analysis of the pup retrieval data. (DOCX)

S2 Table Numbers of mice used in the behavior tests. (XLSX)

S3 Table Real-time PCR primers. (DOCX)
