# Supplementary figures and images for: TDAG51 is a crucial regulator of maternal care and depressive-like behavior after parturition
Source: PLoS Genet. 2019 Jun 28;15(6):e1008214. doi: 10.1371/journal.pgen.1008214 (PMC6599150; doi:10.1371/journal.pgen.1008214)

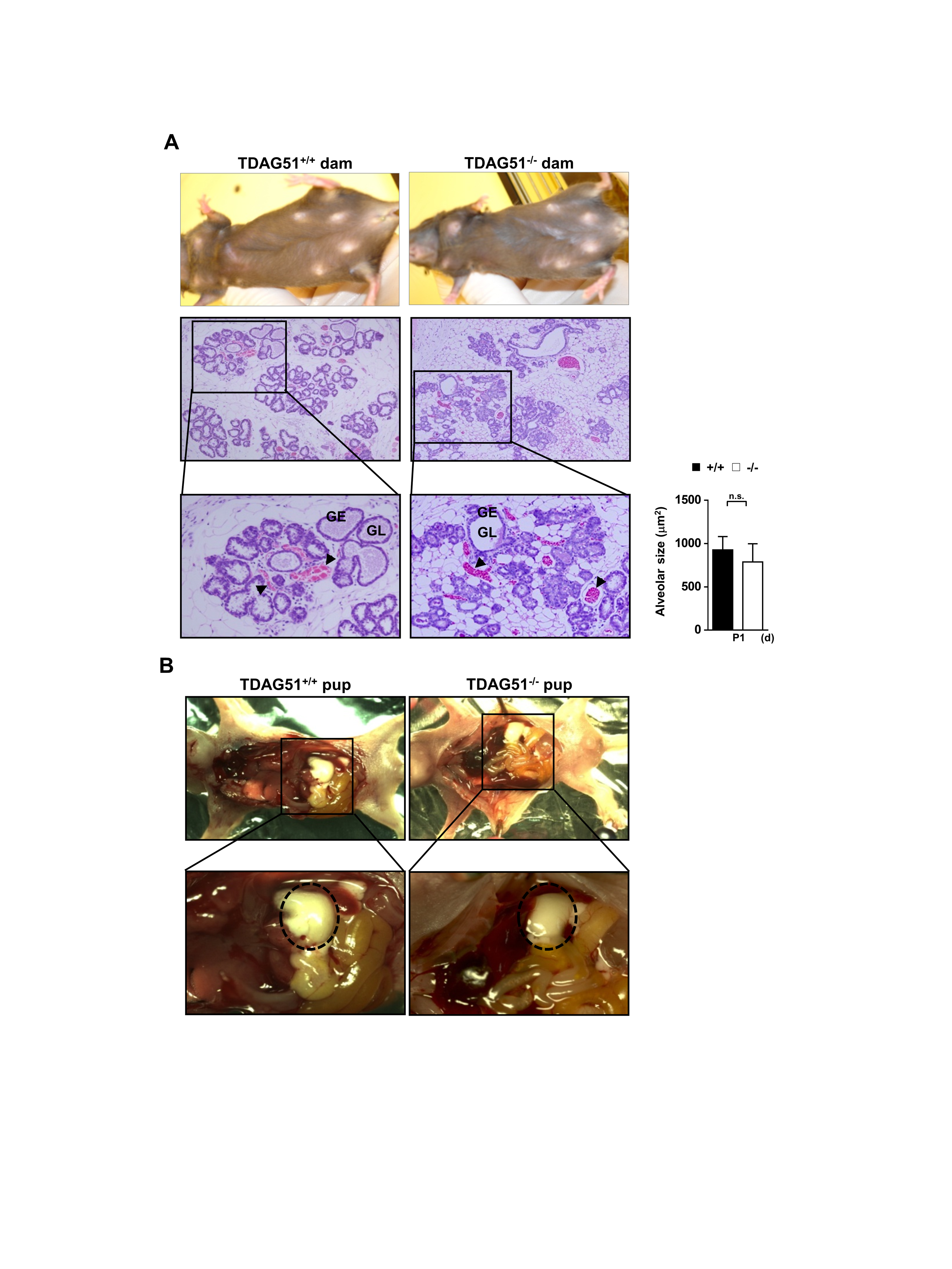

Supplement: S1 Fig — (A) No differences in mammary gland formation and luminal secretion were observed between the TDAG51+/+ and TDAG51-/- dams. The morphology of the mammary papillae (top panel) and histology of the mammary glands (bottom panel) in the TDAG51+/+ dam and TDAG51-/- dam on P1 are shown. The magnified regions are indicated by boxes. GL, glandular lumen. GE, glandular epithelium. Arrows, luminal secretion. The alveolar sizes were compared (right panel). +/+, TDAG51+/+. -/-, TDAG51-/-. n.s., not significant. (B) No suckling problems were observed in the neonate pups. The anatomy of the neonate pups is shown with magnified images. Dotted circles indicate suckled milk in the stomach. (TIF) [file pgen.1008214.s001.tif]

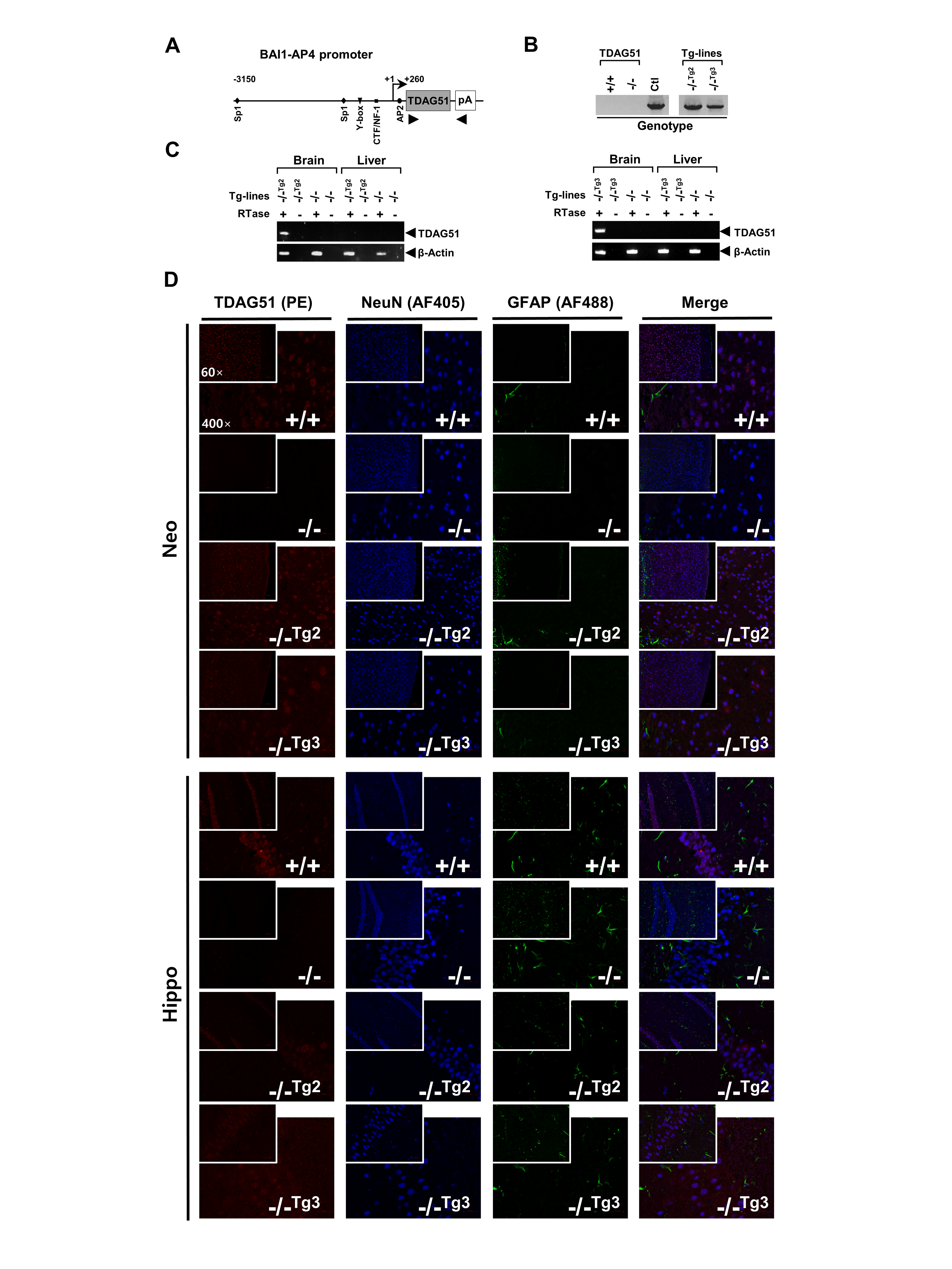

Supplement: S2 Fig — (A) The construction of a brain-specific TDAG51 transgene expression vector. A transgenic vector harboring a brain-specific BAI1-AP4 promoter, the murine TDAG51 gene and an SV40 poly A signal is outlined (left). Nucleotide positions are numbered based on the transcriptional start site, and the putative binding site of the transcription factors is marked on the BAI1-AP4 promoter. Arrows indicate the positions of the genotyping PCR primers. (B) Genotyping of transgenic mice. The transgenic mice genotypes were confirmed by a PCR analysis. +/+, TDAG51+/+. -/-, TDAG51-/-. Ctl, control (TDAG51 transgenic vector). -/-Tg2, transgenic line (Tg-line) 2 on the TDAG51-/- genetic background. -/-Tg3, transgenic mice line 3 on the TDAG51-/- genetic background. (C) TDAG51 expression in the brain of Tg-line mice. TDAG51 expression in the brains of Tg-line mice was compared with that in the livers of Tg-line mice by a quantitative RT-PCR analysis. In the RT control reaction, no reverse transcriptase (RTase) was added. (D) TDAG51 expression was visualized in the brain tissues of the Tg-line mice by an immunofluorescence analysis. Mouse brain tissues were stained with anti-TDAG51 PE-conjugated, anti-GFAP Alexa Fluor 488 (AF488)-conjugated and anti-NeuN Alexa Fluor 405 (AF405)-conjugated antibodies. All images were photographed at a 60× or 400× magnification. Images observed in the same field were merged. Neo, neocortex. Hippo, hippocampus. (TIF) [file pgen.1008214.s002.tif]

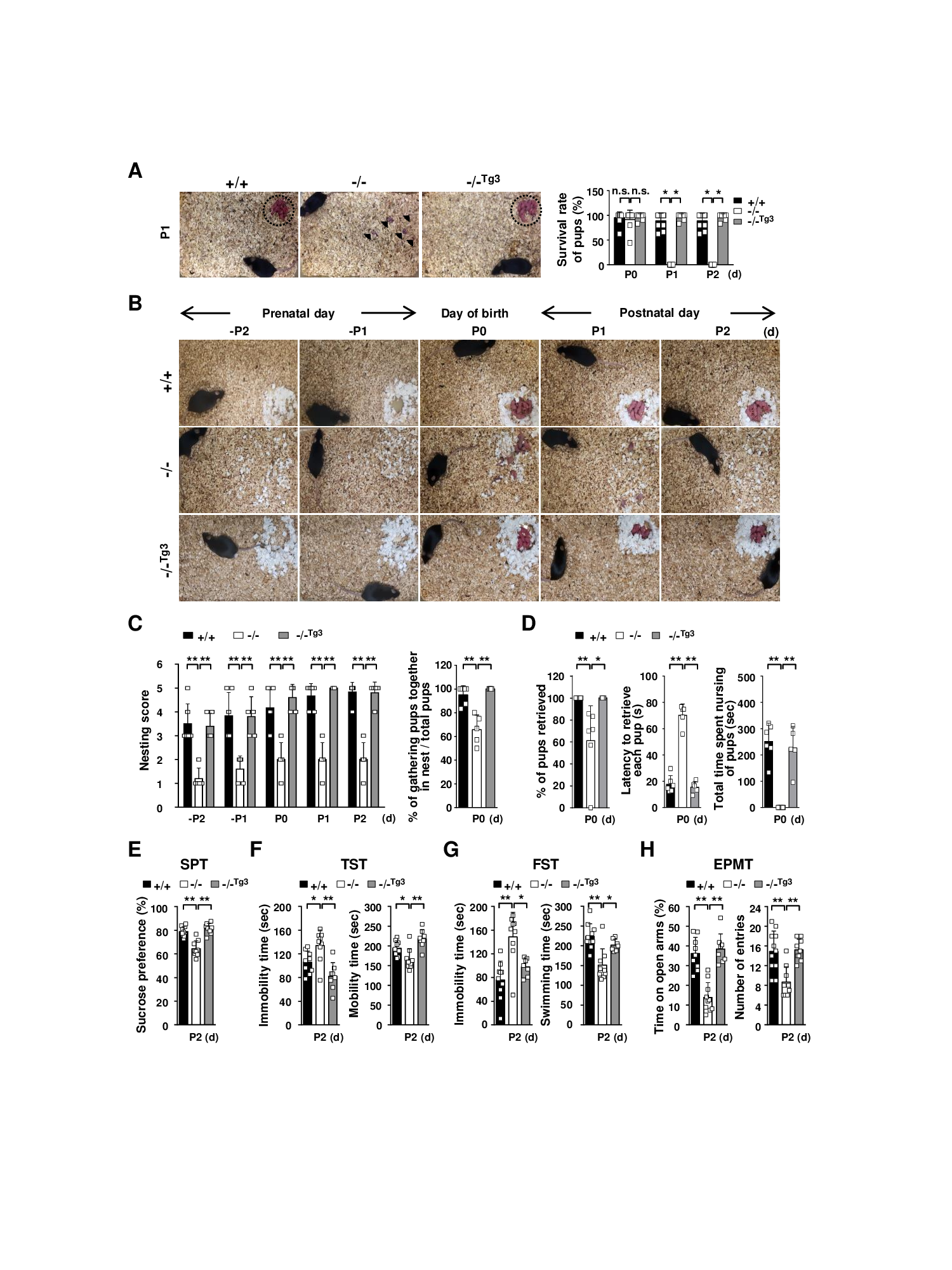

Supplement: S3 Fig — (A) Rescue effect (pup survival test) of brain-specific TDAG51 transgene expression in TDAG51-/- dams. Transgenic line 3 (TDAG51-/-Tg3) mice were generated by expressing TDAG51 in the brain of TDAG51-/- female mice (S3 Fig). The photographs were obtained on P1 (left panel). Arrowheads indicate dead pups. Dotted circles indicate pups gathered in a nest. Survival of pups born to TDAG51+/+, TDAG51-/- and TDAG51-/-Tg3 dams was measured from P0 to P2 (right panel). Black bar (+/+), TDAG51+/+ dams. White bar (-/-), TDAG51-/- dams. Gray bar (-/-Tg3), TDAG51-/-Tg3 dams. (B) Rescue effect (nest building behavior) observed in the TDAG51-/-Tg3 pregnant mice. The photographs of nest building were obtained from -P2 to P2. (C) Measurement of the nest building score. Nesting scores of TDAG51+/+, TDAG51-/- and TDAG51-/-Tg3 pregnant mice (left panel) were analyzed. The number of pups gathered in a nest expressed as a percentage of the total number of neonate pups measured on P0 (right panel). (D) Rescue effect on pup retrieval behavior observed in the TDAG51-/-Tg3 dams. Left panel, the percentage of retrieved pups per dam. Middle panel, latency to retrieve each pup by TDAG51-/- dams. Right panel, impaired nursing of pups by TDAG51-/- dams. (E) SPT. (F) TST. (G) FST. (H) EPMT. *p<0.05. **p<0.01. n.s., not significant. (TIF) [file pgen.1008214.s003.tif]

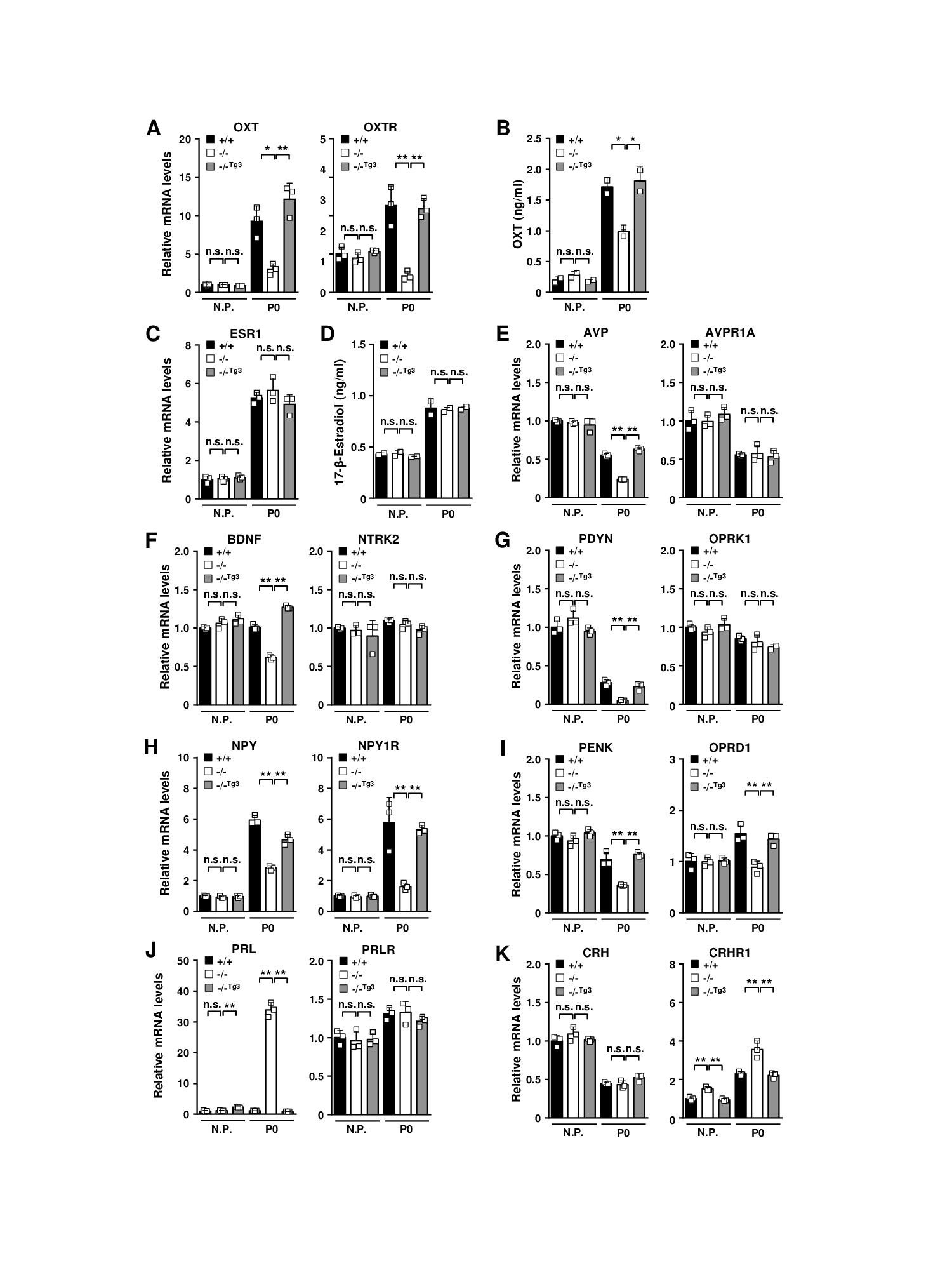

Supplement: S4 Fig — (A) Impaired expression of OXT and its receptor OXTR in TDAG51-/- dams after parturition. Total RNA isolated from brain tissues was analyzed by real-time PCR using specific primers (Table S2). N.P., nonpregnant mice. Black bar (+/+), TDAG51+/+ dams. White bar (-/-), TDAG51-/- dams. Gray bar (-/-Tg3), TDAG51-/-Tg3 dams. (B) Serum OXT levels in TDAG51-/- dams after parturition. Serum OXT levels were analyzed using ELISA. (C) No differences in ESR1 expression were observed in the TDAG51-/- dams after parturition. (D) No differences in serum estrogen levels were observed in the TDAG51-/- dams after parturition. Serum estrogen (17-β-estradiol) levels were analyzed using ELISA. (E) Downregulation of AVP expression in the TDAG51-/- dams after parturition. (F) Downregulation of BDNF expression in the TDAG51-/- dams after parturition. (G) Downregulation of PDYN expression in the TDAG51-/- dams after parturition. (H) Downregulation of the expression of NPY and its receptor NPY1R in the TDAG51-/- dams after parturition. (I) Downregulation of the expression of PENK and its receptor OPRD1 in the TDAG51-/- dams after parturition. (J) Upregulation of PRL expression in the TDAG51-/- dams after parturition. (K) Upregulation of CRHR1 expression in the TDAG51-/- dams after parturition. *p<0.05. **p<0.01. n.s., not significant. (TIF) [file pgen.1008214.s004.tif]

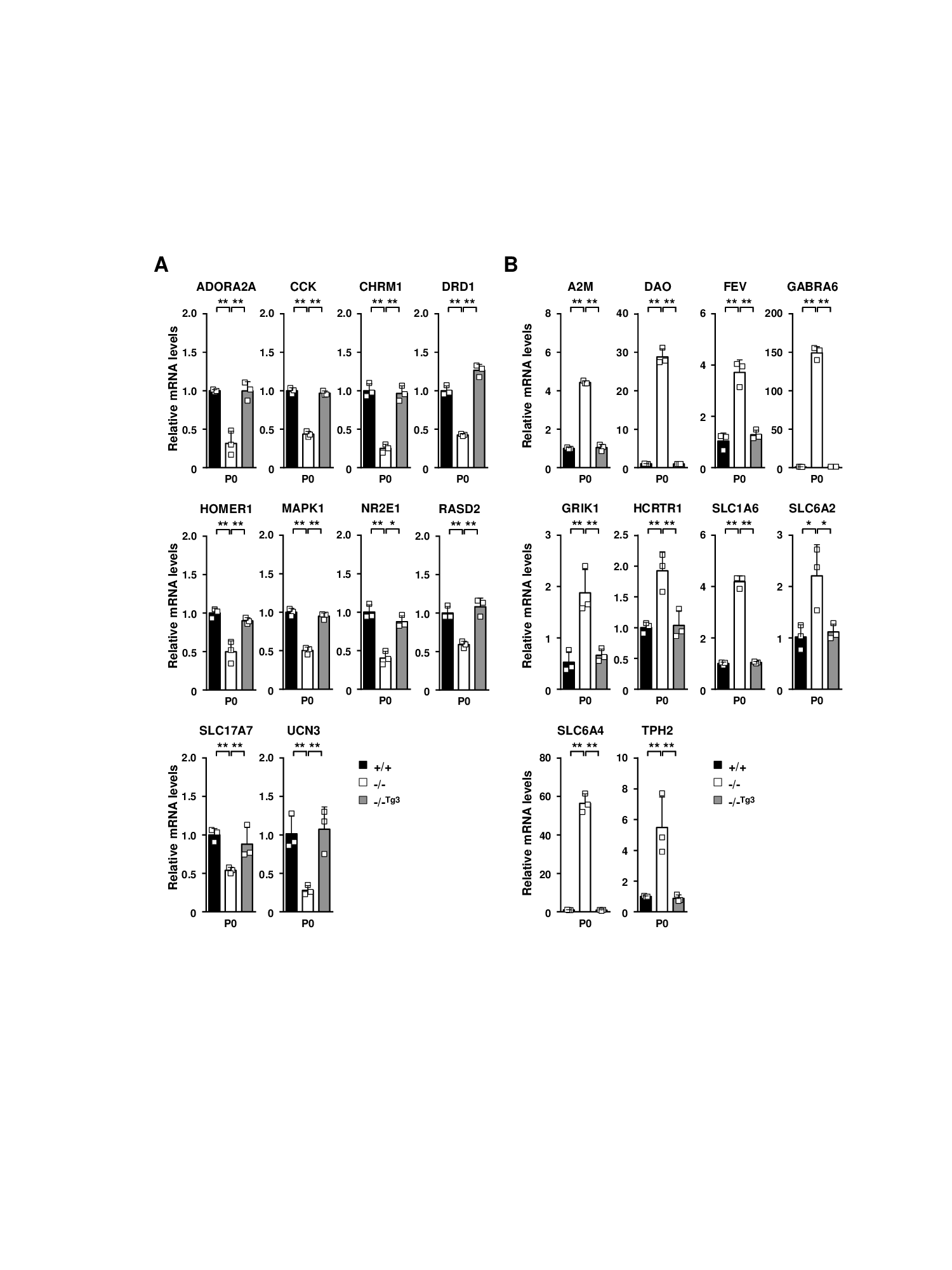

Supplement: S5 Fig — (A) Real-time PCR analysis of downregulated genes selected from the list shown in Table 1. Total RNA isolated from brain tissues was analyzed by real-time PCR using specific primers (Table S2). Black bar (+/+), TDAG51+/+ dams. White bar (-/-), TDAG51-/- dams. Gray bar (-/-Tg3), TDAG51-/-Tg3 dams. (B) Real-time PCR analysis of upregulated genes selected from the list shown in Table 2. *p<0.05. **p<0.01. (TIF) [file pgen.1008214.s005.tif]

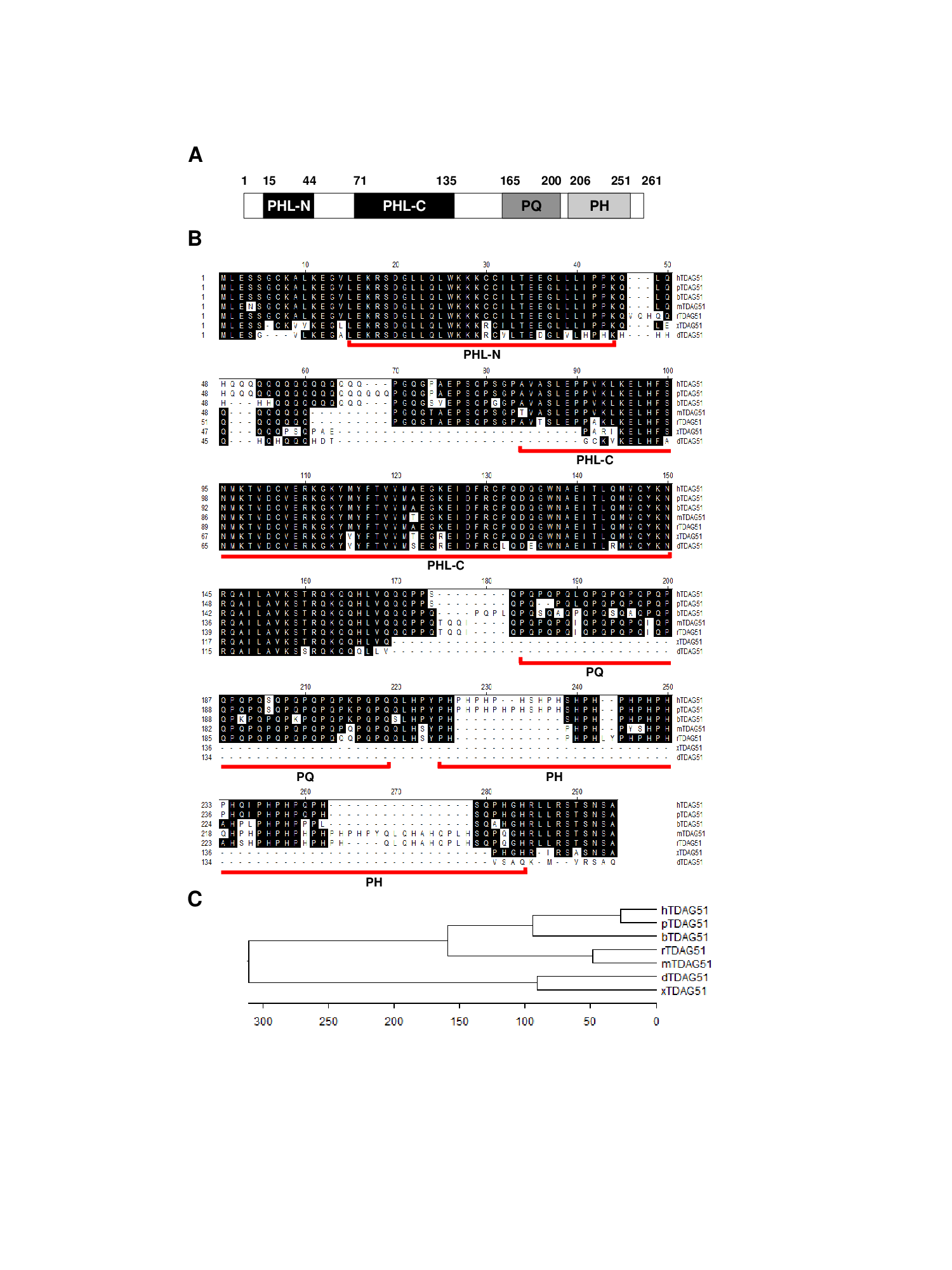

Supplement: S6 Fig — (A) Schematic illustration of the murine TDAG51 domains. The PHL domain, PQ-repeat domain and PH-repeat domain are indicated along with the amino acid residues. PHL-N, N-terminal region of the PHL domain. PHL-C, C-terminal region of the PHL domain. (B) Amino acid sequence alignment of TDAG51 orthologs. Identical residues among the TDAG51 orthologs are shaded in black. Residues of the PHL, PQ and PH domains are marked by a red line. The sequences shown were obtained from the GenBank database under the following codes: hTDAG51 (Homo sapiens, BC110820), pTDAG51 (Pan troglodytes, XM_001161528), bTDAG51 (Bos taurus, BC134549), mTDAG51 (Mus musculus, U44088), rTDAG51 (Rattus norvegicus, AF192802), xTDAG51 (Xenopus laevis, NM_001097698) and dTDAG51 (Danio rerio, NM_001006011). (C) Phylogenetic analysis of the TDAG51 orthologs. The scale bar indicates the nucleotide substitutions per site. (TIF) [file pgen.1008214.s006.tif]
