# Supplementary material for: TDAG51 is a crucial regulator of maternal care and depressive-like behavior after parturition
Source: PLoS Genet. 2019 Jun 28;15(6):e1008214. doi: 10.1371/journal.pgen.1008214 (PMC6599150; doi:10.1371/journal.pgen.1008214)
Supplement: S1 Table — (DOCX) [file pgen.1008214.s007.docx]

**S1Table. Analysis of the pup retrieval data.**

| **Genotype** | **Clone** | **Pup #** | **Retrieved pup #** | **Percentage of pups retrieved** | |  | **Latency to retrieve each pup** | |  | **Total time spent nursing of pups** | |
| --- | --- | --- | --- | --- | --- | --- | --- | --- | --- | --- | --- |
|  |  |  |  | **%** | **Mean±**  **S.E.M. (%)** |  | **Time (s)** | **Mean±**  **S.E.M. (s)** |  | **Time (s)** | **Mean±**  **S.E.M. (s)** |
| **+/+** | #3 | 8 | 8 | 100 | 100±0 |  | 18.7 | 24.8±5.4 |  | 384 | 310±36.1 |
|  | #6 | 7 | 7 | 100 |  |  | 37.6 |  |  | 340 |  |
|  | #15 | 7 | 7 | 100 |  |  | 15.3 |  |  | 312 |  |
|  | #12 | 8 | 8 | 100 |  |  | 38.8 |  |  | 206 |  |
|  | #2 | 6 | 6 | 100 |  |  | 29.8 |  |  | 380 |  |
|  | #8 | 7 | 7 | 100 |  |  | 16.9 |  |  | 211 |  |
|  | #10 | 7 | 7 | 100 |  |  | 16.8 |  |  | 339 |  |
| **-/-** | #3 | 8 | 7 | 87.5 | 57.4±13.7 |  | 63.8 | 63.7±12.3 |  | 0 | 5.0±5.0 |
|  | #2 | 9 | 4 | 44.4 |  |  | 66.7 |  |  | 0 |  |
|  | #13 | 7 | 0 | 0 |  |  | 85.7 |  |  | 0 |  |
|  | #6 | 8 | 6 | 75 |  |  | 75 |  |  | 0 |  |
|  | #5 | 8 | 7 | 87.5 |  |  | 16.1 |  |  | 30 |  |
|  | #11 | 8 | 4 | 50 |  |  | 75 |  |  | 0 |  |

Retrieval rate of pups in TDAG51-/- (-/-) and TDAG51+/+ (+/+) at P0.
