# Supplementary material for: TDAG51 is a crucial regulator of maternal care and depressive-like behavior after parturition
Source: PLoS Genet. 2019 Jun 28;15(6):e1008214. doi: 10.1371/journal.pgen.1008214 (PMC6599150; doi:10.1371/journal.pgen.1008214)
Supplement: S3 Table — (DOCX) [file pgen.1008214.s009.docx]

**S3 Table. Real-time PCR primers.**

| **Gene** | **Primer** | **Sequence** |
| --- | --- | --- |
| **A2M** | Forward | ATG GCC TTT CTT GTG TCC CT |
|  | Reverse | TAA ATG ACG AGG CTG TGC TG |
| **ADORA2A** | Forward | GTC ACC AAC TTC TTC GTG GT |
|  | Reverse | ATG GCG ATG TAT CTG TCG AT |
| **AVP** | Forward | GCA GCA GAT GCT TGG TCC |
|  | Reverse | CTT CCA GAA CTG CCC AAG AG |
| **AVPR1A** | Forward | TTT CGT TTG GAC CGA TTC CG |
|  | Reverse | ATG GGA AGC TCT GGA CAC AA |
| **BDNF** | Forward | GAC GTT TAC TTC TTT CAT GGG C |
|  | Reverse | TGA GTC TCC AGG ACA GCA AA |
| **CCK** | Forward | ATT CGT AGT CCT CGG CAC TG |
|  | Reverse | GCG ATA CAT CCA GCA GGT C |
| **CHRM1** | Forward | CAC ATC CTC TGA AGG TGA GG |
|  | Reverse | AGG TCT TTC TCT TGG CCA GT |
| **CRH** | Forward | ACA GAG CCA CCA GCA GCA T |
|  | Reverse | GGC ATC CTG AGA GAA GTC CC |
| **CRHR1** | Forward | ATG ACC AAA CTC CGA GCA TC |
|  | Reverse | AGA CAC GAA GAA GCC CTG AA |
| **DAO** | Forward | GAT CCA AAA CGA AGC CAT TC |
|  | Reverse | ACC ATT TGG AAG AGC TGC TG |
| **DRD1** | Forward | AGA TGC CGA GGA TGA CAA CT |
|  | Reverse | GTG GTG GTC TGG CAG TTC TT |
| **ERa** | Forward | AAT CTC CAT GAT CAG GTC CA |
|  | Reverse | GCA AAA TGA TGG ATT TGA GG |
| **FEV** | Forward | GGA GAA ACT GCC ACA ACT GG |
|  | Reverse | ACA TGT ACC TGC CAG ATC CC |
| **GABRA6** | Forward | TCT CCC CTG GCT CTT CAT TA |
|  | Reverse | ACA CAG GTC CAA AGC TGG TC |
| **GRIK1** | Forward | AAA AAT CCC TCC GAT CCT GA |
|  | Reverse | CGC GGC ACA GTC CTT ATC C |
| **HCRTR1** | Forward | TTG TGT CTG GCA GAG GAG C |
|  | Reverse | TAG TGT ACG CCA ACA GTG CC |
| **HOMER1** | Forward | ACA TGA GCT CGA GTG CTG AA |
|  | Reverse | AGA CGG AGA AAA TTC CTT GG |
| **MAPK1** | Forward | GGT CTG GTG CTC AAA AGG AC |
|  | Reverse | TCT CGT ACA TCG GAG AAG GC |
| **NPY** | Forward | TGA CCC TCG CTC TAT CTC TG |
|  | Reverse | GTC TCA GGG CTG GAT CTC TT |
| **NPY1R** | Forward | TTC TCC ACT TCT GGC TTT TG |
|  | Reverse | CGA GCA AGT CTG AGA AGG AG |
| **NR2E1** | Forward | GGT AGA TGG GCT AAT TGA CCG |
|  | Reverse | CGC TCT CCA AGA TGA GGC T |
| **OXT** | Forward | CCA GTC TCG CTT GCT GCC TG |
|  | Reverse | GGG CTG CAG CAG ATG CCT GT |
| **OXTR** | Forward | AAG ATG ACC TTC ATC ATT GTT C |
|  | Reverse | CGA CTC AGG ACG AAG GTG GAG GA |
| **PDYN** | Forward | TTA GAG GGC ATC ACG AGG AG |
|  | Reverse | GTC ATT CAT CCC CAG GCT T |
| **PENK** | Forward | TGT TAT CCC AAG GGA ACT CG |
|  | Reverse | TAA ATG CAG CTA CCG CCT G |
| **RASD2** | Forward | TGT TCT CAG CCC AGA GCC AT |
|  | Reverse | AGA CAA TGG AGC TCT TGC CC |
| **SLC17A7** | Forward | CAG GGA GGC TAT GAG GAA CA |
|  | Reverse | AGC ATC TTG ATG GGC ATT TC |
| **SLC1A6** | Forward | CAT GGT AAC CAG TCC TGC CT |
|  | Reverse | GCA GCC ATC TTT ATT GCT CA |
| **SLC6A2** | Forward | CTG ACC ACC ACC ACA AAC AG |
|  | Reverse | ACT CTG CTG GAC ACC TTT GC |
| **SLC6A4** | Forward | GTG ACA GCC ACC TTC CCT TA |
|  | Reverse | CTA GCA AAC GCC AGG AGA AC |
| **SNAP91** | Forward | GGT CTC ATT GGT AGC CTG GA |
|  | Reverse | CAG TAC AGC GTG ACT GGC TC |
| **TPH2** | Forward | TGA GCC CAA GAG ACT TCC TG |
|  | Reverse | CAT CTG AGG CTC CCA GAG AC |
| **NTRK2** | Forward | CAC GAA ACA AGC TGA CGA GT |
|  | Reverse | CGT CAG GAT CAG GTC AGA CA |
| **UCN3** | Forward | GCA CCT CCA GAT CAA AAG AA |
|  | Reverse | GCC TTG TCG ATG TTG AAG AG |
| **β-Actin** | Forward | ATG AAG ATC CTG ACC GAG CG |
|  | Reverse | TAC TTG CGC TGA GGA GGA GC |
